# A Melting Pot of Old World Begomoviruses and Their Satellites Infecting a Collection of *Gossypium* Species in Pakistan

**DOI:** 10.1371/journal.pone.0040050

**Published:** 2012-08-10

**Authors:** Muhammad Shah Nawaz-ul-Rehman, Rob W. Briddon, Claude M. Fauquet

**Affiliations:** 1 Danforth Plant Science Center, St. Louis, Missouri, United States of America; 2 Agricultural Biotechnology Division, National Institute for Biotechnology and Genetic Engineering, Jhang Road, Faisalabad, Pakistan; Naval Research Laboratory, United States of America

## Abstract

CLCuD in southern Asia is caused by a complex of multiple begomoviruses (whitefly transmitted, single-stranded [ss]DNA viruses) in association with a specific ssDNA satellite; Cotton leaf curl Multan betasatellite (CLCuMuB). A further single ssDNA molecule, for which the collective name alphasatellites has been proposed, is also frequently associated with begomovirus-betasatellite complexes. Multan is in the center of the cotton growing area of Pakistan and has seen some of the worst problems caused by CLCuD. An exhaustive analysis of the diversity of begomoviruses and their satellites occurring in 15 *Gossypium* species (including *G. hirsutum*, the mainstay of Pakistan's cotton production) that are maintained in an orchard in the vicinity of Multan has been conducted using φ29 DNA polymerase-mediated rolling-circle amplification, cloning and sequence analysis. The non-cultivated *Gossypium* species, including non-symptomatic plants, were found to harbor a much greater diversity of begomoviruses and satellites than found in the cultivated *G. hirsutum*. Furthermore an *African cassava mosaic virus* (a virus previously only identified in Africa) DNA-A component and a *Jatropha curcas mosaic virus* (a virus occurring only in southern India) DNA-B component were identified. Consistent with earlier studies of cotton in southern Asia, only a single species of betasatellite, CLCuMuB, was identified. The diversity of alphasatellites was much greater, with many previously unknown species, in the non-cultivated cotton species than in *G. hirsutum*. Inoculation of newly identified components showed them to be competent for symptomatic infection of *Nicotiana benthamiana* plants. The significance of the findings with respect to our understanding of the role of host selection in virus diversity in crops and the geographical spread of viruses by human activity are discussed.

## Introduction

Geminiviruses are plant-infecting viruses with circular single-stranded (ss)DNA genomes that are encapsidated in twinned icosahedral (geminate) particles. Based on genome organization, insect vector and host range, the family *Geminiviridae* is classified into four genera: *Begomovirus*, *Curtovirus*, *Mastrevirus* and *Topocovirus* [1], [2]. The genus *Begomovirus* encompasses the majority of the known, as well as economically the most important, geminiviruses that are transmitted exclusively by the whitefly *Bemisia tabaci* [3], [4]. Begomoviruses native to the New World (NW) have genomes consisting of two components, known as DNA-A and DNA-B. Although genetically distinct, bipartite begomoviruses have been identified in the Old World (OW). However, some of the emerging OW begomoviruses are monopartite, consisting of a component homologous to the DNA-A component of the bipartite viruses. Recently it has become clear that the majority of monopartite begomoviruses are associated with betasatellites that are important for infecting some hosts [5].

Both components of bipartite begomoviruses are required for infectivity of plants [6]. The DNA-A component encodes all viral proteins required for replication, control of gene expression and transmission between plants, whereas the DNA-B component encodes two proteins required for intra- and intercellular movement in plants [7]. The integrity of the bipartite genome is maintained by virtue of both components sharing a sequence, known as the common region (CR), which contains the origin of replication (*ori*) [8]. The *ori* consists of the ubiquitous nonanucleotide sequence (TAAT/GATTA/CC) that forms part of a predicted hairpin structure which is nicked by the DNA-A-encoded replication-associated protein (Rep: a rolling circle initiator protein) to initiate replication, and repeated sequence motifs (known as “iterons”), which are sequence specific Rep binding sites and are distinct for each species [9], [10].

Betasatellites are approximately half the size (∼1350 nt) of their helper begomoviruses, which they require for replication and movement in host plants, as well as transmission between plants [5]. Many begomoviruses that associate with betasatellites are wholly dependent on their satellites to efficiently and symptomatically infect some hosts. However, some begomoviruses have a more relaxed relationship, being able to infect plants and induce symptoms in both the presence and absence of the satellite [11]. The mechanism of *trans*-replication of betasatellites by their helper begomoviruses remains unclear. Betasatellites do not encode the iterons of their helper begomoviruses, although they have a predicted hairpin structure (containing a nonanucleotide motif) with similarity to the *ori* of geminiviruses. In most cases betasatellites are capable of being *trans*-replicated by several different begomoviruses. This indicates that their interaction with begomoviruses is distinct from the interaction of DNA-B components with their cognate DNA-A components [12], [13].

Betasatellites encode a single product (known as βC1) that mediates all functions so far ascribed to these molecules. The βC1 protein is a pathogenicity determinant, a suppressor of post-transcriptional gene silencing (PTGS: a host defense mechanism against foreign nucleic acids), possibly binds DNA, may up-regulate viral DNA levels in plants and may provide virus movement functions [14], [15], [16].

Many begomovirus-betasatellite complexes also associate with a further class of satellite-like molecules that are collectively known as alphasatellites. The alphasatellites are also approximately half the size of a typical begomovirus component (∼1380 nt) and encode a single product with similarity to the Rep proteins of another family of ssDNA viruses, the nanoviruses [5], [17]. Alphasatellites are capable of autonomous replication in host plants but require the helper begomovirus for movement in plant tissues and transmission between plants. These molecules apparently perform no essential function for infectivity of plants. However, their almost ubiquitous presence in plants infected with begomovirus-betasatellite complexes suggests they perform some useful, if subtle, function which may provide a selective advantage to the helper begomovirus [5].

Cotton is the major source of fiber and has been produced on the sub-continent since prehistoric times [18]. Fiber in Asia was initially produced from a native cotton species, *Gossypium arboreum*, but is now produced from *G. hirsutum*, which was introduced from Mexico in 1818. Cotton leaf curl disease (CLCuD) was a sporadic problem across southern Asia prior to 1986. In 1986, in the vicinity of Multan (Pakistan), the disease became epidemic and rapidly spread to virtually all cotton growing regions of the country, as well as eastwards into India during the 1990s [19]. During the late 1990s losses due to the “Multan strain” of CLCuD were finally overcome by the introduction of resistant cotton varieties [20]. However, in 2001, resistant cotton varieties in the vicinity of Burewala (Pakistan) began to exhibit symptoms of CLCuD [21]. This signaled the beginning of the second CLCuD epidemic, known as the “Burewala strain”, which now affects all cotton growing areas of Pakistan and northwestern India.

CLCuD in Pakistan during the 1990s was caused by a begomovirus-betasatellite complex that was associated with representatives of at least 6 begomovirus species - *Cotton leaf curl Multan virus* [CLCuMuV], *Cotton leaf curl Rajasthan virus* [CLCuRaV], *Cotton leaf curl Kokhran virus* [CLCuKoV], *Cotton leaf curl Alabad virus* [CLCuAlV], *Papaya leaf curl virus* [PaLCuV] and *Tomato leaf curl Bangalore virus*, either as single or multiple infections [22], [23], [24], [25]. In contrast only a single betasatellite (Cotton leaf curl Multan betasatellite [CLCuMuB]) was isolated [26]. The Burewala epidemic, at least at the present time, is associated with only a single virus, *Cotton leaf curl Burewala virus* (CLCuBuV), which is a recombinant consisting of sequences derived from two of the earlier viruses (CLCuMuV and CLCuKoV) [27]. The betasatellite associated with CLCuBuV is also recombinant, with most of the sequence derived from CLCuMuB [27], [28].

The genus *Gossypium* L. (*Malvaceae*) comprises approximately 50 species of shrubs and small trees which originate from the tropics and sub-tropics. Of these, four species (*G. arboreum* and *G. herbacium*, having diploid genomes and originating from Africa-Asia, and *G. hirsutum* and *G. barbadense*, having tetraploid genomes and originating from the NW) have been cultivated as fiber and oilseed crops for at least 5000 years [29]. It is interesting to note that the native sub-continent species *G. arboreum* is immune to CLCuD, whereas the exotic introduced species *G. hirsutum* and *G. barbadense* are highly susceptible [30]. It is presumed that the native species have had a long association with the viruses causing CLCuD and have evolved resistance.

We have analysed the diversity of begomoviruses (which we shall collectively refer to as “cotton leaf curl geminiviruses” [CGs]) and begomovirus-associated DNA satellites occurring in a collection of mostly non-cultivated *Gossypium* spp. that have been maintained in an orchard in Multan, Pakistan, for over 40 years. The results show the presence of a surprising diversity of components, including new virus species and components, as well as viruses and components that have previously been identified in cotton and other plant species. The significance of these findings to our understanding of the evolution of begomoviruses and the selection pressures exerted upon them by agricultural crops are discussed.

## Results

### Rolling-circle amplification, cloning and sequencing

**Figure 1:**
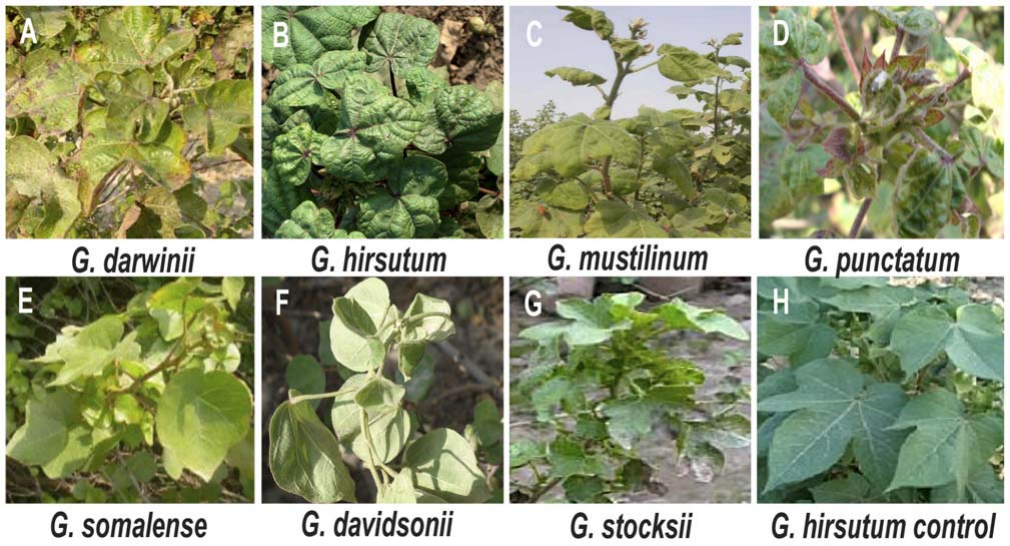
Symptoms displayed by selected *Gossypium* species. Shown are *Gossypium darwinii*, *G. hirsutum, G. mustilinum* and *G. punctatum*, which showed mild to severe symptoms. In contrast, *G. somalense*, *G. davidsonii*, and *G. stocksii* showed relatively mild symptoms. A photo of a healthy *G. hirsutum* plant is shown for comparison.

Leaf samples were collected from symptomatic and asymptomatic plants of the cotton species indicated in Table 1. The symptoms exhibited by plants of selected species are shown in Figure 1 and described in Table 1. DNA was extracted from leaf samples and used to amplify circular DNA molecules using rolling-circle amplification (RCA). RCA yielded a high molecular DNA product from 11 of the 15 *Gossypium* leaf samples. Since RCA exponentially amplifies only circular DNA molecules, the absence of a product for *G. arboreum*, *G. herbaceum* and *G. therburi* is a good indication that these species were not infected with begomoviruses. However, the presence of a high molecular weight DNA product is not necessarily indicative of the presence of a circular DNA virus, since non-viral molecules, such as mitochondrial plasmids, can be amplified by RCA [31]. Following restriction digestion, a total of 34 molecules of ∼2800 nt, 87∼1400 nt and 4 smaller clones were obtained and sequenced in their entirety, in both orientations, with no ambiguities remaining.

**Table 1:** List of *Gossypium* species sampled, symptoms and virus/satellite components identified in each.

						Viruses, virus components and satellites present											
Cotton Species	Geographic Origin	Ploidy level	Agriculturalstatus@	Genome status	Symptoms$	Begomovirus	DNA-B	Betasatellite	ACMV DNA-A	GPMLCuV	CLCuBuV	CLCuMuV	CLCuRaV	CLCuKoV	GDarSLA	GMusSLA	GDavSLA
***G. arboreum***	**Asia**	**Diploid**	**C**	**A**	**NS**	**-**	**-**	**-**	**-**	**-**	**-**	**-**	**-**	**-**	**-**	**-**	
***G. herbaceum***	**Africa**	**Diploid**	**C**	**A**	**NS**	**-**	**-**	**-**	**-**	**-**	**-**	**-**	**-**	**-**	**-**	**-**	
***G. therburi***	**Arizona**	**Diploid**	**W**	**D**	**NS**	**-**	**-**	**-**	**-**	**-**	**-**	**-**	**-**	**-**	**-**	**-**	
***G. davidsonii***	**California**	**Diploid**	**W**	**D**	**M**	**Yes**	**Yes**	**Yes**	**-**	**Yes**	**Yes**	**-**	**-**	**Yes**	**Yes**	**Yes**	**Yes**
***G. gossypioides***	**Mexico**	**Diploid**	**W**	**D**	**VS**	**Yes**	**Yes**	**-**	**Yes**	**Yes**	**Yes**	**-**	**-**	**-**	**-**	**Yes**	
***G. hirsutum***	**Mexico**	**Tetraploid**	**C**	**AD**	**VS**	**Yes**	**-**	**Yes**	**-**	**-**	**-**	**Yes**	**-**	**-**	**Yes**	**-**	
***G. hirsutum***	**Mexico**	**Octaploid**	-+	**AADD**	**VS**	**Yes**	**-**	**Yes**	**-**	**-**	**Yes**	**-**	**-**	**-**	**Yes**	**-**	
***G. barbadense***	**Bolivia, Peru**	**Tetraploid**	**C**	**AD**	**VS**	**Yes**	**-**	**Yes**	**-**	**-**	**-**	**-**	**-**	**-**	**-**	**-**	
***G. darwinii***	**Galapagos Island**	**Tetraploid**	**W**	**AD**	**MS**	**Yes**	**Yes**	**Yes**	**Yes**	**-**	**-**	**-**	**Yes**	**-**	**Yes**	**Yes**	
***G. punctatum*** *	**Mexico**	**Tetraploid**	**W**	**AD**	**S**	**Yes**	**Yes**	**Yes**	**Yes**	**Yes**	**Yes**	**-**	**-**	**-**	**Yes**	**Yes**	
***G. latifolium***	**Mexico**	**Tetraploid**	**W**	**AD**	**MS**	**Yes**	**-**	**Yes**	**-**	**-**	**-**	**-**	**Yes**	**-**	**-**	**Yes**	**Yes**
***G. mustelinum***	**Brazil**	**Tetraploid**	**W**	**AD**	**MS**	**Yes**	**Yes**	**Yes** #	**-**	**-**	**-**	**-**	**-**	**Yes**	**Yes**	**Yes**	
***G. stocksii***	**Pakistan**	**Diploid**	**W**	**E**	**M**	**Yes**	**Yes**	**-**	**Yes**	**-**	**-**	**-**	**-**	**-**	**-**	**-**	
***G. somalense***	**Africa**	**Diploid**	**W**	**E**	**M**	**Yes**	**Yes**	**-**	**Yes**	**-**	**-**	**-**	**Yes**	**-**	**-**	**-**	
***G. lobatum***	**Mexico**	**Diploid**	**W**	**D**	**M**	**Yes**	**Yes**	**-**	**Yes**	**Yes**	**Yes**	**-**	**-**	**-**	**-**	**Yes**	

* A sub-species of *G. hirsutum*.

# Only betasatellite deletion mutants lacking the βC1 gene were identified.

@ Species are indicated as either cultivated (C) or wild (W).

$ Symptoms are indicated as non-symptomatic (NS), mild (M), moderately severe (MS), severe (S) and very severe (VS).

+ Octaploid *G. hirsutum* is non-cultivated, and is maintained for research purposes.

Sequence comparisons showed the ∼2800 nt products to consist of molecules with similarity to the genomes (or DNA-A components) and DNA-B components of begomoviruses. The ∼1400 nt clones were shown to have similarity with beta- and alphasatellites. The presence of each type of molecule in the individual leaf samples of plants each of the *Gossypium* species is indicated in Table 1.

### Diversity of begomoviruses in the *Gossypium* species

The complete sequences of 34 potentially full-length molecules, originating from 10 non-cultivated cotton species and one cultivated species (*G. hirsutum*), homologous to the DNA-A components of bipartite begomoviruses were obtained. The features of these sequences and the accession numbers under which they are available in the databases are given in Table S1 and S2.

**Figure 2:**
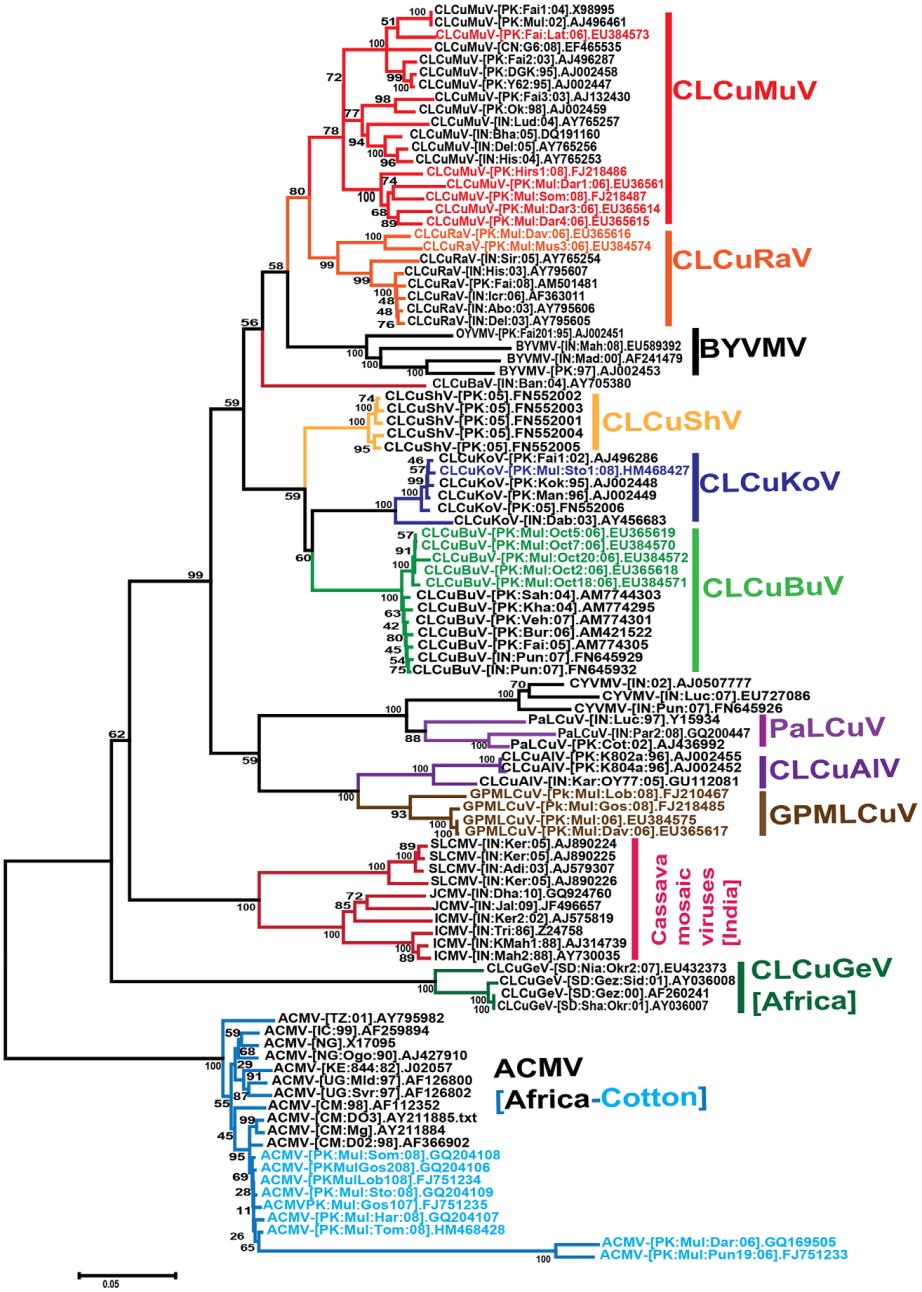
Phylogenetic analysis of virus genome (or DNA-A component) sequences. Neighbor-joining dendrogram based upon a Clustal W alignment all begomovirus genome (or DNA-A component) sequences determined here with sequences of the genomes (or DNA-A components) of selected begomovirus species occurring in the Old World. The database accession number of each sequence is given. The acronyms and isolate descriptors are as described in Fauquet *et al.* [1]. The numbers at nodes represent percentage bootstrap values (1000 replicates).

The begomovirus genome (or DNA-A component) sequences obtained were used in a phylogenetic analysis based upon an alignment with the full-length genome (or DNA-A) sequences of selected begomovirus isolates available from the databases, representing the majority of begomovirus species so far identified in cotton on the Indian subcontinent (Figure 2) [1]. The tree shows the majority of the sequences obtained here to segregate with previously identified virus species which have been shown to infect cotton in southern Asia, specifically CLCuMuV, CLCuRaV, CLCuBuV and CLCuKoV. The assignment of each sequence to a specific species is indicated in Table S1. Pairwise sequence comparisons confirmed the assignment of each clone to a species based on the presently applicable species demarcation threshold for begomoviruses (89%; Table 2) [32].

**Table 2:** Highest and lowest percentage nucleotide sequence identity values for pairwise comparisons of the begomovirus genomes (DNA-A components) with selected sequences available in the GenBank database.

Sequences obtained from the databases*											
Sequences determined here#	CLCuMuV (12)	CLCuRaV (6)	CLCuAlV (4)	CLCuKoV (6)	CLCuBaV (1)	CLCuBuV (6)	PaLCuV (4)	GPMLCuV (−)	CLCuShV (5)	ACMV (11)	ICMV (6)
CLCuMuV (6)	97-86	91-83	83-76	84-72	83-77	85-74	71-60	84-77	89-79	65-61	69-64
CLCuRaV (2)		99-95	79-74	89-74	81-80	80-74	71-61	84-75	75-73	64-60	68-65
CLCuAlV (4)			94-89	68-66	75-73	71-66	73-67	87-81	74-71	62-59	64-62
CLCuKoV (1)				99-89	80-77	88-82	80-72	70-65	93-84	66-62	70-68
CLCuBaV (1)					-	79-75	69-63	75-71	82-81	65-63	70-69
CLCuBuV (5)						100-97	76-69	72-68	88-86	66-63	70-67
PaLCuV (4)							100-85	75-67	74-68	64-57	67-60
GPMLCuV (4)								100-91	75-73	60-57	64-60
CLCuShV (5)									99-98	66-64	70-68
ACMV (9)**										99-92	65-62
ICMV (6)											98-89

* The figure in brackets indicates the numbers of sequences used in the analysis.

# The species highlighted (white text on a black background) are the sequences determined here. The remaining species are sequences from the databases included for comparison.

In addition to the isolation of begomoviruses species previously identified in cotton, the phylogenetic tree showed an unusual group of isolates (indicated as “GPMLCuV” in Figure 2). These isolates are most closely related to, and segregate with, CLCuAlV. They were isolated from four cotton species; *G. davidsonii*, *G. gossypioides*, *G punctatum* and *G. lobatum*. The four clones show between 91 and 100% nucleotide sequence identity (the clones from *G. davidsonii* and *G punctatum* having identical sequences), but less than 87% identity to all other geminivirus sequences available in the databases. The highest levels of sequence identity were to isolates of CLCuAlV (between 81 and 87%) and CLCuMuV (between 77 and 84%; Table 2). To isolates of CLCuRaV available in the databases, the sequences showed only between 75 and 84% identity (Table 2). Based on these results the four clones represent isolates of a new begomovirus species, for which we propose the name *Gossypium punctatum mild leaf curl virus* (GPMLCuV).

Surprisingly the DNA-A component of *African cassava mosaic virus* (ACMV), a bipartite begomovirus not previously identified in Asia, was identified in six of the cotton species (Table 1). However, no evidence for the presence of ACMV DNA-B was found, either by PCR amplification with specific primers or by Southern blot hybridization (results not shown). A total of 9 potentially full-length ACMV DNA-A clones were obtained and sequenced (Table S2). The sequences of these clones show between 76% and 99% identity. To the sequences of ACMV isolates available in the databases they show between 92% and 99% identity, with the highest levels of identity to an isolate originating from Cameroon (ACMV-[CM:03], AY211884). The predicted amino acid sequences of each of the gene products showed the ACMV isolates from Pakistan to have the highest identity levels to ACMV isolates originating from Cameroon and Ivory Coast (results not shown). Many of the ACMV DNA-A clones obtained are defective, containing frame-shift mutations of the virion-sense genes (as detailed in Figure S1) but with the complementary sense containing only very few single nucleotide exchanges. For many of the clones it is unlikely, even if the cognate DNA-B were present, that they would be able to infect plants autonomously, suggesting that they are maintained by *trans*-complementation.

### Recombination between begomoviruses in *Gossypium* species

**Figure 3:**
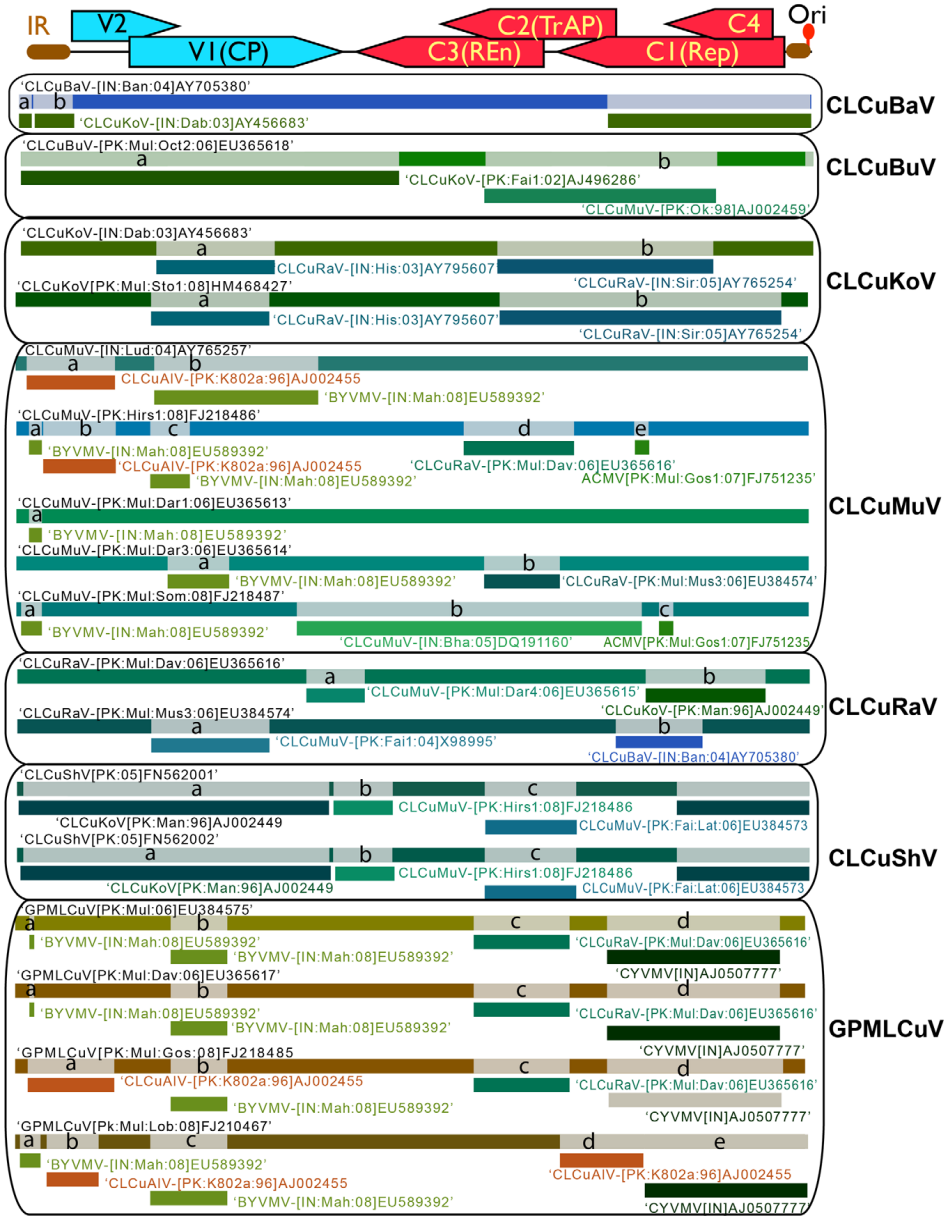
Analysis for recombination among selected begomoviruses associated with CLCuD. Recombinant fragments in the sequences of CLCuD-associated begomoviruses were identified using RDP3 analysis of a Clustal W alignment. The default X-Over settings was used for recombination analysis. Recombinant sequences are identified as colored lines below the sequence (identified above each full-length sequence line) together with its origin (parent). The sequences analysed are grouped according to species (marked on the right). A liner genome map (with the position of genes and their orientation indicated with arrows; blue for virion-sense and red for complementary-sense) is shown at the top of the figure to indicate the positions of recombinant fragments. The intergenic region (IR; blown bar) and hairpin structure of the *ori* are shown.

Recombination is a common feature in the evolutionary history of many geminiviruses [33], [34]. To determine whether the begomoviruses identified here show evidence of recombination, RDP3 analysis was conducted based on alignments with full-length sequences of selected begomoviruses available in the databases. The results of this are shown in Figure 3 with the details, including p-values, given in Table S3. The analysis showed CLCuBuV to consist of the virion-sense sequences of CLCuKoV and the complementary-sense sequences of CLCuMuV (Figure 3), as reported previously [27].

Recombination analysis for CLCuMuV clones obtained here shows that each host in which this virus was identified harbors isolates with distinct recombination patterns. Isolates from *G. hirsutum* ([PK:Hir1:08], FJ218486), *G. somalense* ([PK:Mul:Som:08], FJ218487) and *G. darwinii* ([PK:Mul:Dar1:06]) were overall very similar, with sequences just downstream of the hairpin-loop apparently originating from *Bhendi yellow vein mosaic virus* (BYVMV), although the isolate [PK:Hir1:08] also contains a fragment derived from CLCuAlV and resembles an isolate originating from India ([PK:K802a:96]). Interestingly, isolates [PK:Hir1:08] and [PK:Mul:Som:08] contain a recombinant fragment originating from ACMV (Figure 3). However, *G. hirsutum* was not found to harbor ACMV, indicating that either the recombination event yielding CLCuMuV-[PK:Hir1:08] occurred in another plant/species, with the virus subsequently being transmitted to *G. hirsutum*, or that the *G. hirsutum* plant earlier contained ACMV but has since lost it.

CLCuRaV was identified in two cotton species, *G. davidsonii* and *G. mustilinum*, and these two sequences showed differing recombination patterns. In contrast, CLCuKoV, which was only identified in a single cotton species (*G. stocksii*), showed 99% nucleotide sequence identity to previously characterized CLCuKoV isolates, indicative of little, if any, recombination having occurred since these isolates diverged.

GPMLCuV is unique to the orchard in Multan, having not so far been identified elsewhere, and was isolated from 4 cotton species (*G. punctatum, G. lobatum*, *G. davidsonii* and *G. gossypioides*). This virus may thus have evolved in this orchard; a hypothesis that is supported by the high levels of sequence conservation (91 to 100% identity) between isolates obtained from distinct cotton species (Table 2). Recombination analysis showed the sequences of GPMLCuV to exhibit incongruous segregation (Figure 3). For three isolates ([PK:Mul:06], [PK:Mul:Dav:06] and PK:Mul:Gos:06]) the coat protein (CP) and V2 genes showed recombination with BYVMV. However, the V2 gene GPMLCuV isolates [PK:Mul:Gos:08] and [PK:Mul:Lob:08] showed recombination with CLCuAlV. For the Rep gene all isolates exhibited recombination with *Croton yellow vein mosaic virus* (CYVMV). For the TrAP and REn genes all isolates except [PK:Mul:Lob:08] showed recombination with CLCuRaV. Isolate [PK:Mul:Lob:08] instead contained an additional CLCuAlV fragment in the Rep/TrAP gene overlap region.

Overall the recombination analysis shows that, with the possible exception of CLCuKoV, the viruses identified here have a highly recombinant origin which is distinct from, in most cases, the viruses identified earlier. The analysis also shows that viruses with quite distinct histories of recombination can make up a single begomovirus species. This is one of the drawbacks of a taxonomy that includes, as a major criterion, sequence relatedness.

### Identification of a begomovirus DNA-B component in *Gossypium* species

No bipartite begomoviruses infecting cotton have previously been reported from the OW. Thus it came as a surprise that molecules with similarity to the DNA-B components of bipartite begomoviruses were isolated from 8 cotton species (Table 1). Despite an extensive search, however, no DNA-B component was identified in *G. hirsutum*, consistent with previous studies [19], [23], [27].

The eight DNA-B molecules obtained showed between 88 and 98% nucleotide sequence identity, showing them to be closely related, despite each having been isolated from a different cotton species. Comparisons to sequences available in the databases showed only relatively low percentage identity values to the sequences of DNA-B components of other begomoviruses originating from the OW (<50%) with the exception of DNA-B components of *Sri Lanka cassava mosaic virus* (SLCMV; 65–69%), *Indian cassava mosaic virus* (ICMV; 65–71%) and the recently identified *Jatropha curcas mosaic virus* (JCMV; 68–71%) [35](Table 3). This suggests that the DNA-B components identified in cotton originate from one of these three virus species or a species closely related to these viruses.

**Table 3:** Highest and lowest percentage identity values for pairwise comparisons of the complete nucleotide sequences of the DNA-B components of selected begomoviruses with the DNA-B components identified in cotton species.

	GPMLCuV (8)*	ICMV (6)*	SLCMV (3)*	JCMV (1)*	ToLCNDV (5)*
**GPMLCuV (8)** *	98-88	71-65	69-65	71-68	35-32
**ICMV (6)** *	-	98-75	94-91	82-81	34-32
**SLCMV (3)** *	-	-	98-95	79-78	33-32
**JCMV (1)** *	-	-	-	100	36-33
**ToLCNDV (5)** *		-	-	-	97-83

* The figures in brackets indicate the numbers of isolates compared.

**Figure 4:**
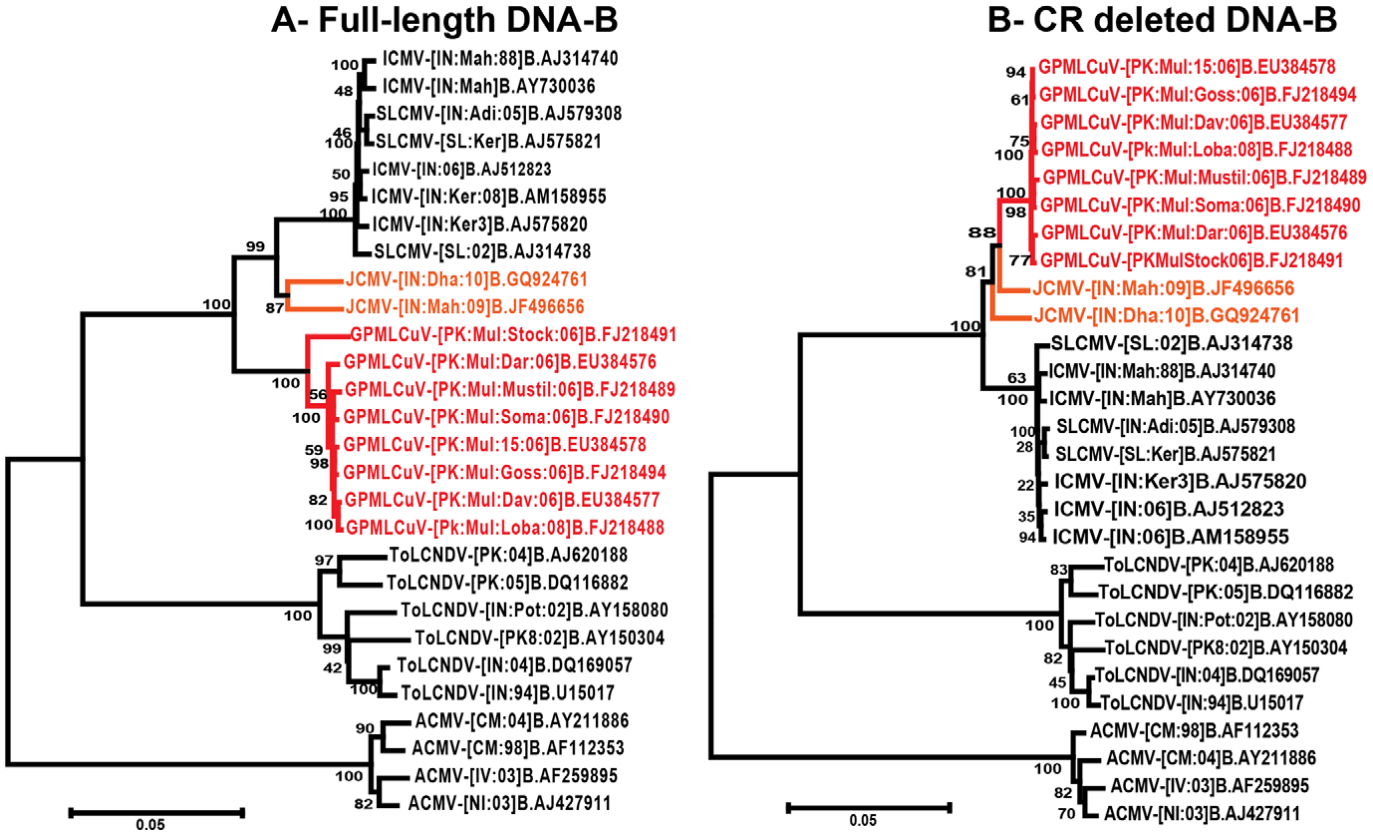
Phylogenetic analysis of DNA-B component sequences. Neighbor-joining dendrogram based upon a Clustal W alignment of all DNA-B components determined here with sequences of selected DNA-B components of begomovirus species occurring in the Old World. The tree in panel A is based upon full-length sequences whereas that in panel B is based upon sequences spanning the BC1 and BV1 region. The database accession number of each sequence is given. The acronyms and isolate descriptors are as described in Fauquet *et al.* [1]. The numbers at nodes represent percentage bootstrap values (1000 replicates).

A phylogenetic tree, based upon an alignment of all available DNA-B sequences with the sequences of DNA-B obtained here (Figure 4A), shows the cotton components to be most closely related to, but distinct from, the DNA-B components of ICMV, JCMV and SLCMV. It has previously been shown that the DNA-B components of ICMV and SLCMV have a common origin, most likely due to component exchange between the two begomovirus species [36]. Although for the most part consisting of the sequence of ICMV DNA-B, the SLCMV DNA-B contains the *ori* of SLCMV, allowing the SLCMV DNA-A-encoded Rep to *trans*-replicate this DNA-B component. To ascertain if a similar exchange of the *ori* has occurred in the cotton DNA-B components, a phylogenetic tree based upon the DNA-B sequences without the CR sequences was produced (Figure 4B). This shows the JCMV DNA-B sequences to segregate with, and be basal to, the cotton DNA-B sequences and both to be distinct from the ICMV and SLCMV DNA-B components. Additionally the DNA-B components of ICMV and SLCMV co-segregate, supporting the conclusion of Saunders *et al.* [36] that they have a common origin.

Alignments of the GPMLCuV sequences with their cognate DNA-B sequences showed them to contain a shared sequence spanning the *ori*. For example, GPMLCuV-[PK:Mul:Dav:06] (EU365617) and its cognate DNA-B (EU384577) share a sequence of ∼280 nt (coordinates 2594 to 144 and 2567 to 142, respectively) that has 91.5% identity; overall the sequences show only 46% identity. This is good evidence of *ori* donation. This is supported by the fact that all the DNA-B isolates share the same predicted iteron sequences (GGGGA) that are also found in GPMLCuV (the presumed donor of the *ori* sequences in the DNA-B molecules isolated from cotton), CLCuAlV and CYVMV. In contrast, the predicted iterons of JCMV (the presumed parent of most of the sequences making up the cotton DNA-Bs), ICMV and SLCMV are GGTA, whereas those of CLCuKoV are GGTA/G.

Interestingly, all the cotton DNA-B sequences contain a unique duplication of the right (3′) leg of the nonanucleotide-containing stem-loop structure (Figure S2). The significance of these duplications and whether they might play a part in component replication is unclear.

### Betasatellites identified in *Gossypium* species

The presence of betasatellites was shown in 7 of the 14 cotton species examined (Table 1) and the complete sequences of 27 presumed full-length molecules were obtained (Table S4). The molecules showed between 86 and 99% nucleotide sequence identity, indicating that they are all isolates of a single species of betasatellite (based upon the proposed species demarcation threshold of 78% for betasatellites) [37]. With the exception of isolate [PK:Mul:Lat11:06] and 5 clones from *G. davidsonii*, all are of the typical size of betasatellites (∼1350 nt) being between 1349 and 1359 nt in length. These presumed full-length betasatellites have the conserved structure shown previously for this class of satellites, consisting of a single gene (βC1), a region of sequence rich in adenine (A-rich) and a sequence of ∼100 nt conserved between all betsatellites, known as the satellite conserved region (SCR) [26]. CLCuMuB-[PK:Mul:Lat11:06], (EU384591) is an unusual mutant with a perfect inverted duplication (coordinates 259–744) repeating 243 nt of the βC1 coding sequence which replaces the N-terminal end of the *bona fide* βC1 gene, the βC1 promoter and much of the A-rich sequence.

**Figure 5:**
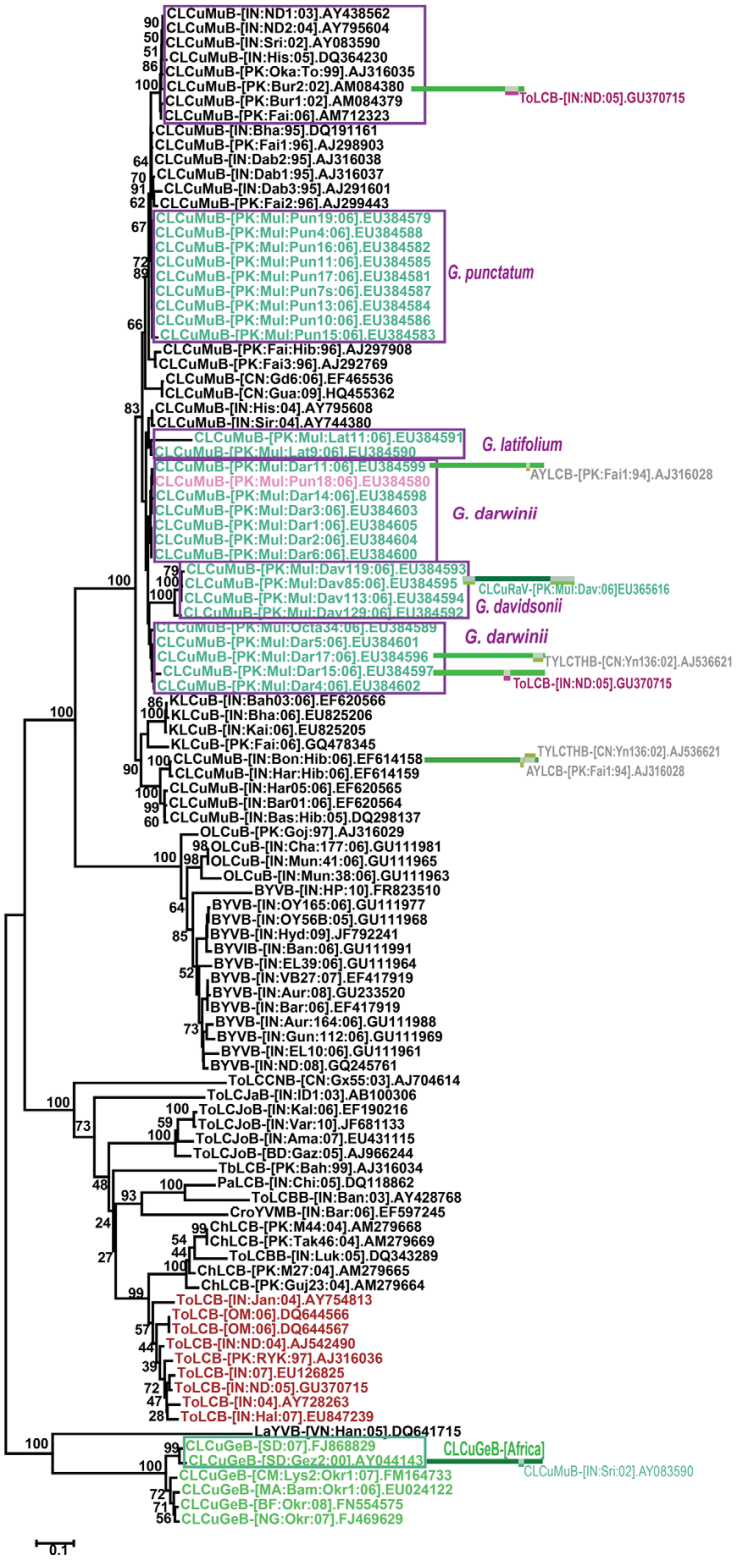
Phylogenetic analysis of betasatellite sequences. Neighbor-joining dendrogram based upon a Clustal W alignment of selected betasatellite sequences available in the databases with the betasatellite sequences determined here (a more complete phylogenetic analysis is shown in Figure S3). The database accession numbers of each sequence are given. The acronyms and isolate descriptors are as described in [37]. The betasatellite isolates obtained from cotton spp. as part of this study are highlighted with colored boxes. The numbers at each node represent the bootstrap values (1000 replicates). The recombinant sequences for different hosts are presented in different rectangles. The representative sequences of recombinant origin are presented with their line diagram generated through RDP3 program. Note that some betasatellite species in this tree are not monophyletic due to the use of the Clustal W algorithm for the alignment (the taxonomy of betasatellites is based upon the Clustal V algorithm).

A phylogenetic tree, based upon an alignment of all betasatellite sequences available in the databases with the betasatellite sequences obtained here is shown in Figure S3, whereas a more compact tree with fewer sequences is shown in Figure 5. Both trees show the betasatellites to fall into the two major phylogenetic classes first identified by Briddon *et al.*, (2003); those isolated, for the most part, from hosts in the family *Malvaceae*, and those originating mostly from non-malvaceous hosts. The betasatellites identified here all fall into the malvaceous class and segregate into 4 distinct groups. The first group, consisting of four sequences isolated from *G. davidsonii*, are recombinant, with the SCR replaced by the intergenic sequence derived from CLCuRaV ([PK:Mul:Dav129:06]), or CLCuMuV ([PK:Mul:Dav118:06], [PK:Mul:Dav113:06] and [PK:Mul:Dav85:06]). Such recombinant betasatellites have been identified previously in association with CLCuD affected cotton [19] and other begomovirus-betasatellite complexes [38], [39] and are, by convention, not classified as betasatellites [37].

The second group of betasatellite molecules (isolated from *G. punctatum*) segregates with the first CLCuMuB identified, the betasatellite associated with CLCuD in Pakistan during the 1990s [19], which we shall henceforth refer to as CLCuMuB^Mul^. The third group (isolated from *G. darwinii*) is closely related to CLCuMuB^Mul^ but contains an approximately 95 nt sequence, within the SCR, derived from Tomato leaf curl betasatellite (ToLCuB). This betasatellite, which we shall refer to as CLCuMuB^Bur^, is associated with CLCuBuV and is the only satellite now prevalent in *G. hirsutum* in most cotton growing areas of Pakistan [27], [28]. Interestingly, previously isolated CLCuMuB^Bur^ sequences from Pakistan and India form a cluster that is distinct from CLCuMuB^Bur^ sequences isolated from *G. darwinii*. This may be due to the shorter length of ToLCuB sequence inserted in these CLCuMuBs. Among the betasatellites isolated from *G. darwinii* there are betasatellites ([PK:Mul:Dar17:06], EU384596) which are recombinant with Tomato yellow leaf curl Thailand betasatellite (TYLCTHB) and Ageratum yellow leaf curl betasatellite (AYLCB; [PK:Mul:Dar11:06], EU384599) rather than ToLCB. So far, TYLCTHB has not been identified in Pakistan (Figures 5 and S3).

The fourth group of betasatellites, consisting of two clones isolated from *G. latifolium* (CLCuMuB-[PK:Mul:Lat9:06] and -[PK:Mul:Lat11:06]), do not contain the recombinant SCR of CLCuMuB^Bur^ and have sequences between the SCR and A-rich region that are distinct from both CLCuMuB^Bur^ and CLCuMuB^Mul^, the origin of which remains unclear. Five further CLCuMuB clones, three of which were cloned from *Hibiscus* spp. [40], [41], [42], segregate with four Kenaf leaf curl betasatellite (KLCuB) isolates which together are basal to all CLCuMuB sequences. A possible explanation is that these CLCuMuB and KLCuB clones were isolated from the far-east Indian state of West Bengal (along the Bangladesh border), and thus are geographically isolated from the areas where CLCuD occurs. Additionally these CLCuMuB clones are also recombinant, containing sequences in the SCR derived from AYLCB and TYLCTHB.

Recently the African CLCuD-associated begomovirus *Cotton leaf curl Gezira virus* (CLCuGeV) has been identified in cotton originating from southern Pakistan in the presence of CLCuMuB [25]. The cognate betasatellite of CLCuGeV, Cotton leaf curl Gezira betasatellite (CLCuGeB), has not so far been identified in Pakistan. The recombination analysis here has shown a small recombinant fragment of CLCuMuB in a CLCuGeB isolate from Sudan ([SD:Gez2:00], AY044143) (Figure 5). This suggests that CLCuMuB may be present in Africa and further highlights the exchange of viruses and associated satellites between Africa and southern Asia.

*G. lobatum*, *G. gossypoidies*, *G. stocksii* and *G. somalense*, despite containing viruses previously classified as betasatellite-requiring monopartite geminiviruses (CLCuBuV or CLCuRaV), did not apparently contain betasatellites. In contrast, only a betasatellite derived from CLCuMuB^Bur^, with the βC1 gene deleted, was identified in *G. mustilinum* (data not shown). This is the first such molecule derived from CLCuMuB^Bur^, all previous βC1deletion mutants having been derived from CLCuMuB^Mul^.

### Alphasatellites identified in *Gossypium* species

Alphasatellites were identified in 8 of the 14 *Gossypium* species (Table 1). A total of 60 full-length alphasatellite molecules were cloned and sequenced (Table S4). These range from 1141 to 1373 nt in length, typical of this class of molecule [17]. All the molecules contain a single large gene which encodes a Rep protein homologous to those of previously characterized alphasatellites.

**Figure 6:**
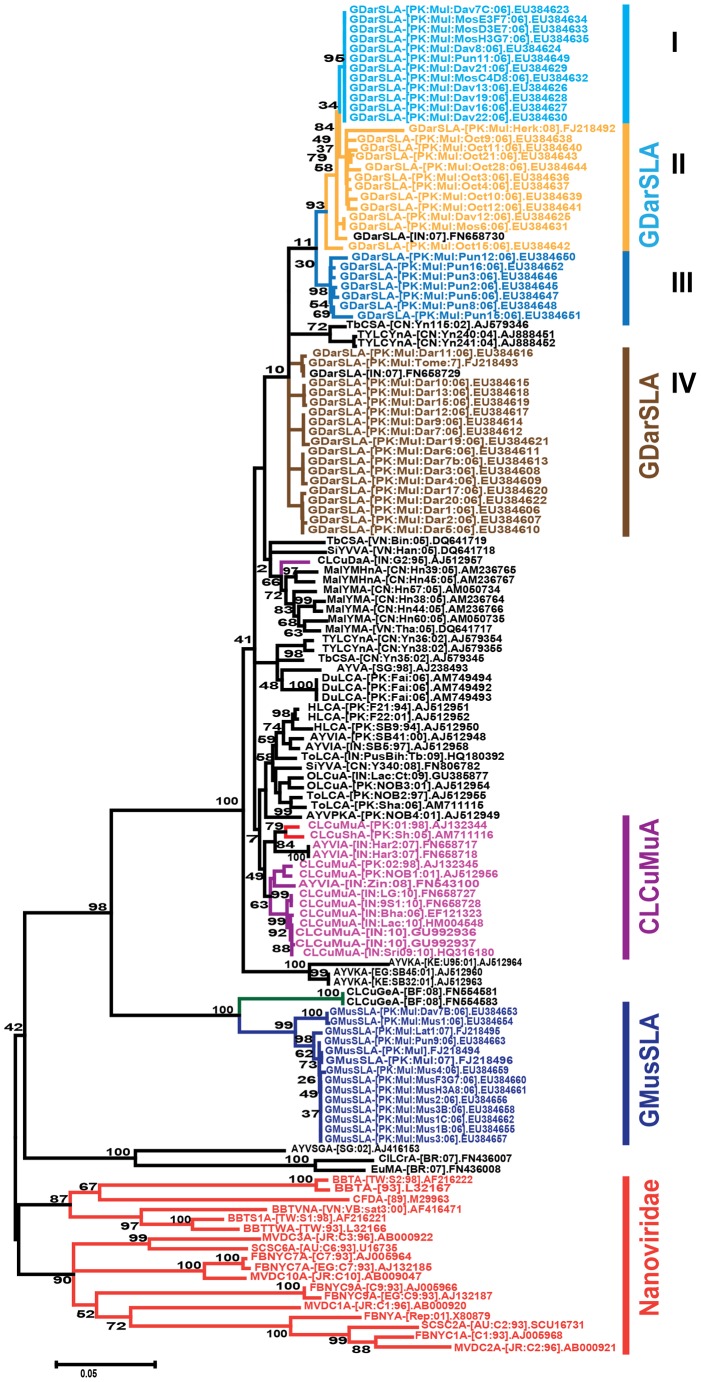
Phylogenetic analysis of alphasatellite sequences. Neighbor-joining dendrogram based upon a Clustal W alignment of the alphasatellite sequences determined here with selected alphasatellite sequences available in the databases. The database accession numbers of each sequence is given. The alphasatellite isolates obtained from cotton (including those not isolated in this study) are highlighted with colored text and indicated on the right. The alphasatellites labeled I to IV are discussed in the text. The alphasatellites associated with nanoviruses are indicated with red text and red branches. The numbers at nodes represent percentage bootstrap values (1000 replicates). Note that some alphasatellite in this tree are not monophyletic due to the use of the Clustal W algorithm for the alignment (the taxonomy of alphasatellites is based upon the Clustal V algorithm).

A phylogenetic tree, based upon an alignment of all full-length alphasatellite sequences available in the nucleotide sequence databases with the full-length alphasatellite sequences obtained here is shown in Figure 6. This shows the alphasatellites characterized here to fall into three major groups (indicated as “GMusSLA”, “GDarSLA” and “GDavSLA”). The alphasatellites identified here are distinct from the two alphasatellite so far identified in cotton in Pakistan; Cotton leaf curl Multan alphasatellite (CLCuMuA) and Cotton leaf curl Shadadpur alphasatellite (CLCuShA) [17], [43], [44], [45].

The 49 alphasatellites labeled as GDarSLA show between 85 and 100% nucleotide identity with each other (Table 4). To all other alphasatellites available in the databases the GDarSLA sequences show less than 67% identity, with the exception of CLCuMuA to which the identity values are between 75 and 86%. Based on the proposed demarcation threshold for distinct aplhasatellites (83%; R.W. Briddon, manuscript in preparation), this indicates that they represent isolates of a single alphasatellite, for which we propose the name Gossypium darwinii symptomless alphasatellite (GDarSLA).

**Table 4:** Highest and lowest percentage identity values for comparisons of the complete nucleotide sequences of the alphasatellites identified here with selected alphasatellites associated with begomoviruses and nanoviruses obtained from the databases.

Sequences determined in this study			Sequences obtained from the databases				
	GMusSLA (14)*	GDarSLA (51)* [95]#	CLCuMuA (9)* [99-86]@	CLCuGeA (2)* [100]@	ToLCA (3)* [94-80]@	FBNYVC9A◊ (2)* [98]@	BBTA◊ (2)* [92.3]@
GMusSLA (14)*[100-88]▴	-	57-52	56-54	57-51	52-44	41-38	46-45
GDarSLA (49)*[100-85]▴	-	-97-89	78-75	37-33	67-56	40-32	32-24

* The figures in round brackets indicate the numbers of isolates compared.

@ The figures in square brackets are the percentage identity values for comparisons between the sequences obtained from the databases.

# There are two GDarSLA sequences available in the databases, originating from India. The figure in brackets is the percentage identity between these two sequences.

▴ The figures in square brackets are the percentage identity values for comparisons between the sequences obtained from *Gossypium* spp.

◊ Two nanovirus-associated alphasatellites are included for comparison; Faba bean necrotic yellow vein C9 alphasatellte (FBNYVC9A) and Banana bunchy top alphasatellite (BBTA).

The alphasatellites labelled as GMusSLA, which were isolated from 6 cotton species (Table S5) form a clade with Cotton leaf curl Gezira alphasatellite (CLCuGeA; note that this alphasatellite has so far not been identified in cotton). The GMusSLA sequences share between 88 and 100% nucleotide sequence identity with each other but show less than 57% identity to all other alphasatellites, the highest being to CLCuGeV (51–57%) and Tomato leaf curl alphasatellite (44 to 52%; Table 4). This indicates that they represent a new alphasatellite for which the name *Gossypium mustilinum symptomless alphasatellite* (GMusSLA).

GMusSLA is distinct from all other begomovirus-associated alphasatellites in encoding a Rep consisting of only 295aa with the exception of isolate [PK:Mul:Gos-2:08], that contains a number of frame shift mutations yielding a gene with the potential to encode a 302aa product and isolates ([PK:Mul:Dav7b:06], (EU384653) and [PK:Mus1:06], (Eu384654) that instead encompass a gene with the capacity to encode a shorter product of 263aa due to a frame shift mutation that C-terminally truncates the coding sequence. Isolates [PK:Mul:Dav7b:06] and [PK:Mus1:06] are significantly smaller (1218 nt) than typical begomovirus-associated alphasatellites (∼1380 nt), which may indicate that they are deletion mutants. With the exception of the alphasatellite described by Saunders et al., [46] AYVSGA, (previously named DNA-2), which has recently also been identified in Oman [47], that encodes a 289aa Rep, other full-length begomovirus-associated alphasatellites typically encode a Rep consisting of a predicted 315aa.

Interestingly AYVSGA and the alphasatellites identified in the NW (Cleome leaf crumple alphasatellite [CILCrA] and Euphorbia mosaic alphasatellite [EuMA] [48], are basal to all the other begomovirus-associated alphasatellites and the GMusSLA/CLCuGeA clade sits between these two groups. This indicates that there are three distinct classes of begomovirus-associated alphasatellites, which are only distantly related to the nanovirus alphasatellites.

### Dating estimates for the origins of CGs

**Figure 7:**
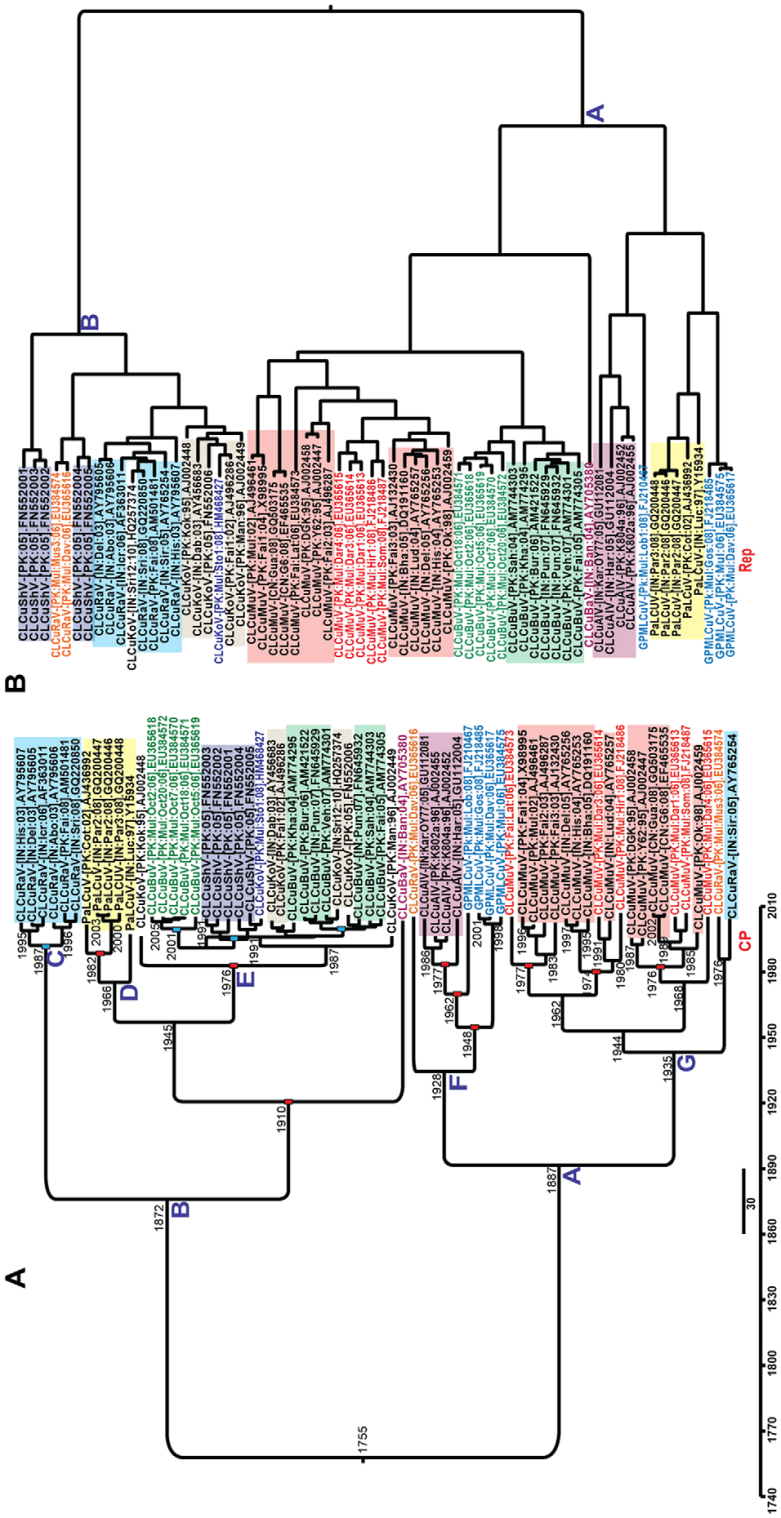
Dating estimates for the origins of CGs. Dated phylogeny for the coat protein (CP) genes of CLCuD-associated begomoviruses originating from Asia (A). The scaled to time trees were generated by using uncorrelated relaxed LogNormal clock model in the BEAST program (v1.6). The base of each clade is labeled with the mean time to most recent common ancestor (TMRCA) values. Red boxes on nodes indicate viruses which were present before the first appearance of CLCuD in 1967, while blue boxes represent viruses which appeared after the first incidence of the disease. A non-dated phylogeny for the replication-associated protein of CLCuD-associated begomoviruses originating from Asia is shown for comparison (B). Due to recombination the lineages in panel B do not the mirror those of panel A and recombination also leads to some virus species not being monophyletic.

To estimate when CGs associated with CLCuD first emerged, time to most recent common ancestor (TMRCA) was estimated from phylogenies of the CP gene using Markov Chain Monte Carlo (MCMC) integrated in BEAST (V1.6). By applying the relaxed uncorrelated relaxed clock model with exponential growth parameters, the dates for CGs lineages were estimated (Figure 7A). An undated tree, based alignments of the Rep gene sequences, was produced for comparison (Figure 7B).

Interestingly, both trees shown in Figure 7 have two major clades (labeled A and B). In both trees clade A contains CLCuMuV, while the clade B encompasses CLCuKoV. Not surprisingly the phylogenetic tree generated for Rep gene sequences shows CLCuBuV to form a monophyletic group with CLCuMuV, while for the CP tree CLCuBuV segregates with CLCuKoV isolates, confirming the conclusions of Amrao et al. [27] and the recombination analysis here concerning the origins of this species. For the CP tree GPMLCuV segregates with CLCuAlV which form a clade segregates with PaLCuV isolates, indicating the possible emergence of GPMLCuV by recombination between CLCuAlV and PaLCuV. It is possibly significant that CLCuAlV, PaLCuV and GPMLCuV form a distinct group in the Rep tree, suggesting that for the Rep sequences they diverged from the remainder of the cotton viruses some time ago. It is also worth noting that only on one occasion has PaLCuV been identified in cotton [23]. Overall these trees show that, for at least some of the CGs, recombination is a major feature in their origins.

For the CP tree the date estimates have broad confidence intervals (Table S6), indicating that caution needs to be taken in interpreting the divergence time estimates. The CP sequences of nine divergent CGs [Taxa n = 66]) isolated between 1995 and 2010 were used to estimate their times of emergence. The data indicates that CGs could be ∼2.5 centuries old and that a major diversification of CGs could have started during the late 19^th^ century (Figure 7A, nodes A and B). CLCuAlV/GPMLCuV and CLCuMuV are estimated to have appeared as long ago as ∼1935 and 1928 (nodes F and G), whereas PaLCuV and CLCuKoV are estimated to have diverged in 1945. CLCuD was first noted in Pakistan in 1967 [49]. The TMRCA distribution for DNA-A component shows that 6 out of 9 CGs were potentially co-circulating at that time. However, CLCuBuV and CLCuShV (node E) seem to appear due to recombination after the mid-1980s. In contrast, the nodes for the divergence of GPMLCuV (node F) show that this recombinant is older (mean value 1948) than CLCuBuV. However, since 2001 only CLCuBuV has been found in *G. hirsutum* across most of Pakistan, while GPMLCuV has so far only been identified in the orchard in Multan. This may indicate that GPMLCuV is not well adapted to *G. hirsutum* or at least that CLCuBuV is better adapted.

### Dating estimates for the origins of satellites

**Figure 8:**
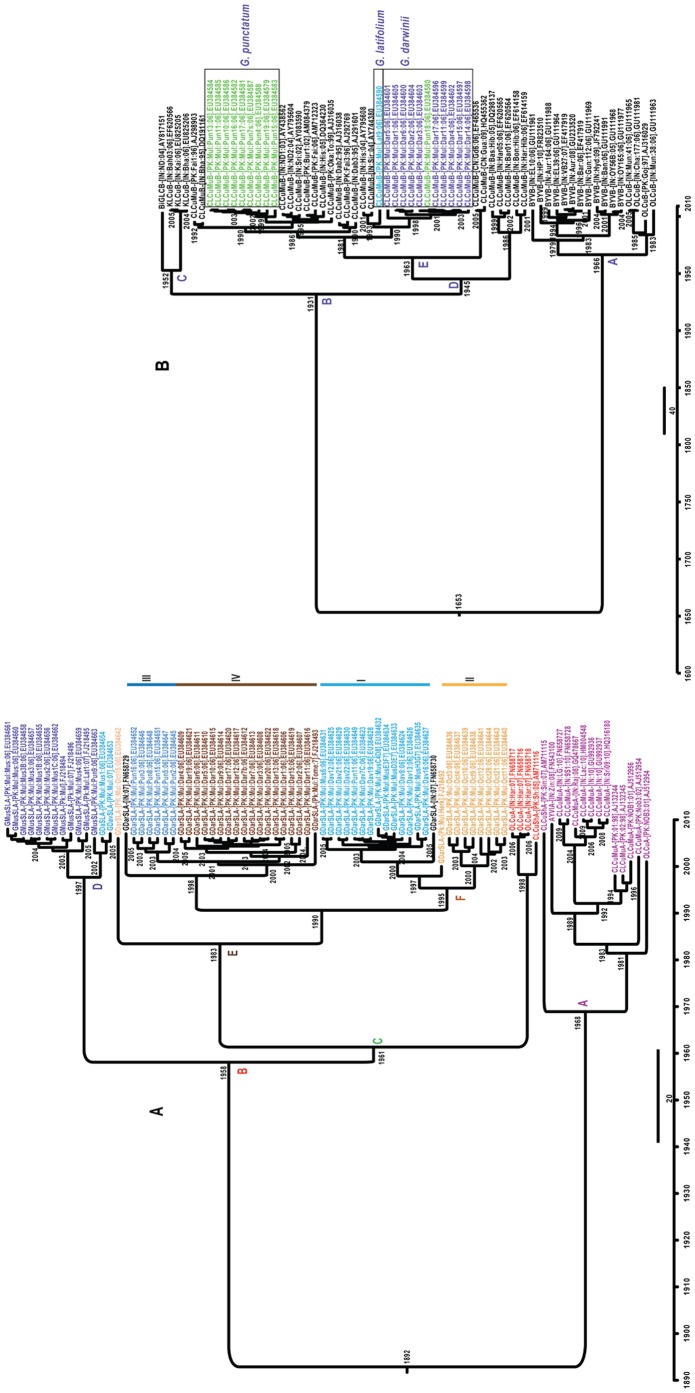
Dating estimates for the origins of cotton leaf curl begomovirus-associated satellites. Dated phylogeny of the Rep (panel-A) and βC1 (panel-B) proteins of alphasatellites and betasatellites, respectively, associated with CLCuD. The sequences were selected on the basis of prior information from pairwise comparisons and phylogenetic trees presented in Figures 5 and 6. The trees were automatically rooted through uncorrelated relaxed LogNormal clock model in BEAST program (V1.6). The years of divergence are shown on bases of each node. For panel A the groups as sequences labeled I to IV are discussed in the text. For panel B the cotton species from which betasatellites obtained here were isolated are shown on the right.

Due to betasatellites in some cases being recombinant (Figure 5), which might influence the results in the maximum clade credibility (MCC) tree, the βC1 gene (∼357 nt) was selected for divergence time estimates. Similarly, due to differences in the sequence lengths of alphasatellites, the Rep gene of alphasatellites (alpha-Rep) gene was used for TMRCA estimates. The TMRCA distributions (Figure 8A and B) suggest that the cotton-associated alphasatellites emerged (mean value 1852) more recently than the cotton-associated betasatellite (mean value 1653). The oldest possible date for the emergence of CLCuMuA is 1968 (clade A; Figure 8A), when it diverged from its closest relative CLCuShA (AM711116). Interestingly, CLCuMuA isolates from Pakistan and India form separate groups within clade A. This indicates that alphasatellites isolated from these countries are evolutionarily isolated. Importantly, of the alphasatellites in the clade B (Figure 8A), GMusSLA forms a distinct group and has the oldest TMRCA estimates (mean value 1958). It is noteworthy that GDarSLA isolates group according to the host from which they were isolated with the exception of those in group I. Group-I isolates originate from *G. davidsonii* and *G. mustilinum*. This is similar to the pattern observed for the full-length genomes of alphasatellites (Figure 6) and may suggest that the GDarSLA groupings reflect host adapted variants and/or that there is little exchange between the cotton species, with the exception of *G. davidsonii* and *G. mustilinum*.

TMRCA estimates for the βC1 gene of OLCuB, CLCuMuB, and KLCuB (clades A, B and C; Figure 8B) show the same branching pattern as the phylogenetic trees based on full-length sequences (Figure 5). CLCuMuB shares a more recent common ancestor with KLCuB than either does with the betasatellites that infect okra; Bhendi yellow vein betasatellite (BYVB) and Okra leaf curl betasatellite (OLCuB)(node A: Figure 8B). The data suggests that a major diversification of CLCuMuB occurred after 1963, which is just 4 years before CLCuD was first reported in Pakistan. The independent grouping of CLCuMuB isolates from *G. punctatum* and *G. darwinii* suggests that there is little exchange of the betasatellite with these two species.

### Estimation of nucleotide substitution rates for CGs and their satellites

The mean nucleotide substitution rates for the CP of CLCuMuV, the βC1 gene of CLCuMuB and the Rep gene of GDarSLA were determined using recombination free datasets with the relaxed clock and Bayesian Skyline Plot (BSP) method (Table 5). For each dataset, the sequences were partitioned into the 3 codons positions. The mean substitute rates for βC1 and the alphasatellite Rep were considerably higher (3.51×10^−3^ and 2.13×10^−3^ substitutions/nucleotide/year, respectively) than those for the CLCuMuV CP (4.24×10^−4^). This high substitution rate is closer to substitution rate estimated for the *East African cassava mosaic virus* CP (1.37×10^−3^ subst./nt/year), a bipartite cassava infecting begomovirus from Africa [50]. The high nucleotides substitution rate for satellites suggests that they are evolving rapidly. This idea is supported by the fact that satellite clones isolated from a single host segregate within the phylogenetic trees. The substitution rate for the CLCuMuV CP (4.24×10^−4^ subst./nt/year) is similar to that estimated for that estimated for the *Tomato yellow leaf curl virus* CP (4.63×10^−4^ subst./nt/year). This may indicate that these two viruses face the same evolutionary pressure, despite infecting different hosts in different parts of the world. The differences between mutation rates for CP and the satellite genes also indicate that these are likely under different evolutionary (selection) pressures.

**Table 5:** Mean substitution rates for satellites encoded proteins and CLCuMuV-encoded coat protein.

			Mutation rate	Mutation rate	Mutation rate	
Model used	Gene	Number of sequences used	Codon position 1	Codon position 2	Codon position 3	Mean rate (substitutions/nucleotide/year)
Relaxed clock+BSP	CLCuMuB βC1	39	.85	0.73	1.43	3.51×10^−3^
Relaxed clock+BSP	GDarSLA Rep	63	1.4	0.765	0.831	2.13×10^−3^
Relaxed clock+BSP	CLCuMuV CP	19	1.64	0.449	0.909	4.24×10^−4^
Relaxed clock+BSP*	EACMV CP	71	-	-	-	1.37×10^−3^
Relaxed clock+exponential**	TYLCV CP	54	-	-	-	4.63×10^−4^

* Duffy and Holmes, 2009.

** Duffy and Holmes, 2008.

To further estimate the selection pressure, we used the 3 position clock model in BSP analysis. Surprisingly, for the GDarSLA Rep and the CLCuMuV CP, but not the CLCuMuB βC1, codon position 1 showed a higher substitution rate (1.4 and 1.64, respectively) than codon positions 2 and 3 (0.765, 0.831 and 0.449, 0.909, respectively). This is unexpected since the third codon position (wobble position) normally shows a higher rate of substitution. This likely indicates that the βC1 gene is under a higher selection pressure, preventing sequence change, than the other two genes. Why the CP and alpha-Rep genes might show more rapid sequence change is unclear. Sequence changes (particularly at codon 1) would usually be considered detrimental. For the CP this might interfere with insect transmission, so the data may indicate that GDarSLA is no longer under the stringent selection pressure posed by insect transmission (the viruses possibly no longer requiring insect transmission, since the plants are maintained vegetatively). This cannot, however, be the case for GDarSLA Rep, since mutations here would interfere with replication of the satellite and could lead to extinction. Further studies will be required to investigate this phenomenon.

### GPMLCuV can transreplicate both a betasatellite and a DNA-B in *Nicotiana benthamiana*

**Figure 9:**
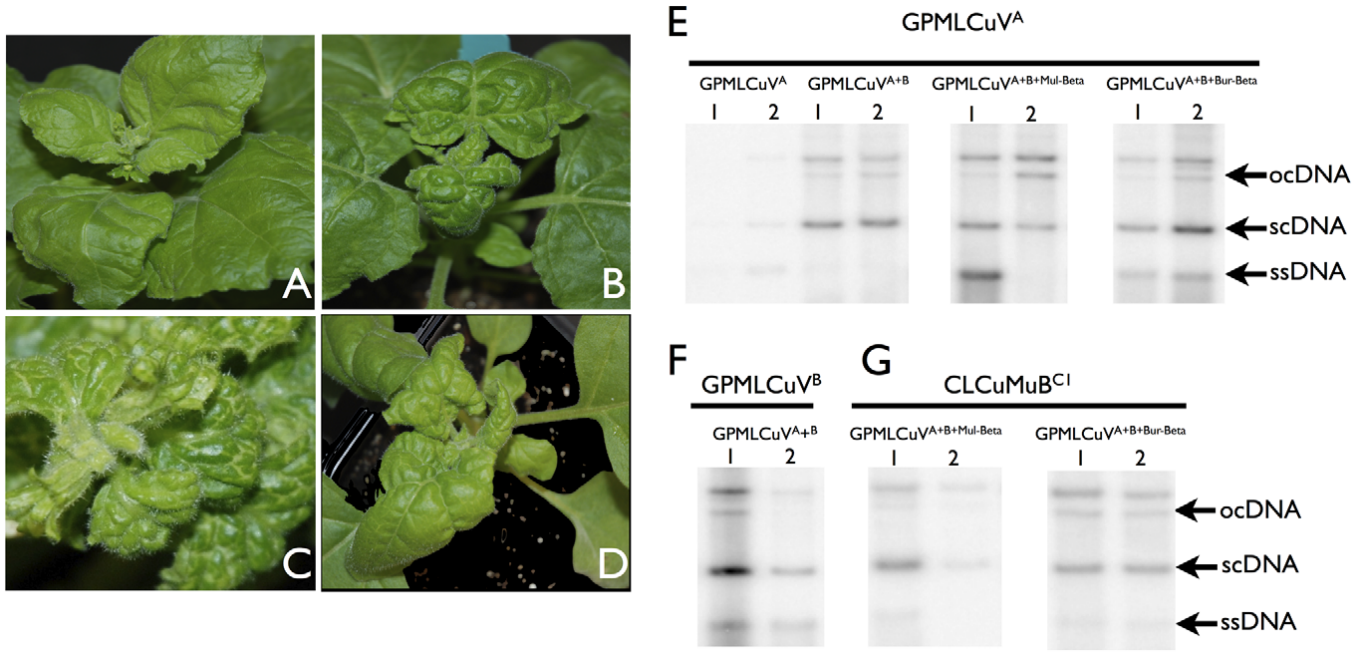
Infectivity of GPMLCuV to *N. benthamiana* in the presence DNA-B and CLCuMuB. *N. benthamiana* plants were inoculated with GPMLCuV (GPMLCuV^A^), GPMLCuV with its cognate DNA-B (GPMLCuV^A+B^), GPMLCuV with its cognate DNA-B and CLCuMuB^Mul^ (GPMLCuV^A+B+Mul-Beta^), and GPMLCuV with its cognate DNA-B and CLCuMuB^Bur^ (GPMLCuV^A+B+Bur-Beta^). Plants were photographed at 21 dpi. Southern hybridization for each combination is shown in panels E, F and G. In each case, approximately, 0.5 ug of DNA isolated from two different plants was loaded (lanes 1 and 2) on agarose gels and transferred to nylon membranes. Panel E was probed with the C4 gene of GPMLCuV. Panels F and G were probed with the BV1 and βC1 genes of DNA-B and CLCuMuB respectively. The replicative DNA forms are indicated as single-stranded (ss), supercoiled (sc) and open-circular (oc).

Biolistic inoculation of GPMLCuV to *Nicotiana benthamiana* resulted in very mild leaf curl symptoms at 14 days post-inoculation (dpi)(Figure 9, panel A). At 21 dpi newly developing leaves showed increasingly milder symptoms and plants recovered from infection (results not shown). Co-inoculation of GPMLCuV ([PK:Mul:Dav06], EU365617) with the cognate DNA-B component ([PK:Mul:Dav06], EU384577) resulted in severe downward leaf curling and vein thickening symptoms at 14 dpi (panel B). However, no recovery was observed for these infections. Interestingly, inoculation of GPMLCuV with either CLCuMuB^Mul^ or CLCuMuB^Bur^ resulted in very severe symptoms (panels C and D, respectively), including leaf enations and infertility of flowers. Symptoms in the presence of either betasatellite were more severe than in the presence of the DNA-B. For each of the inoculations, 10 plants were inoculated and all showed symptoms of infection.

Southern blot analysis showed that in *N. benthamiana* plants GPMLCuV is capable of maintaining both CLCuMuB and the DNA-B component (Figure 9, panels E–G). Infections of GPMLCuV in the presence of the DNA-B raised viral DNA levels above those seen in plants infected with only the virus. However, there was no significant difference in viral DNA levels between infections with either CLCuMuB^Mul^ or CLCuMuB^Bur^, in the presence of the DNA-B. Both betasatellite variants were efficiently maintained by the virus in *N. benthamiana*.

## Discussion

Annual crops such as cotton are re-infected with geminiviruses every growing season from sources that must include other crops and weeds, as well as volunteer (ratoon) cotton. These plants act as reservoirs of both the viruses and insect vectors in the off season. This annual cycle between crop and reservoir hosts is a stringent bottleneck that potentially reduces the genetic diversity of the viruses in the crop. Each year only the best adapted viruses survive the bottleneck and spread within the crop. It is only recently that researchers have come to realize that weeds may harbor a far greater diversity of viruses and their satellites, than actually appears in the crop, and that it is possible that genetic changes (including for example component exchange and recombination) within weeds plays a major role in virus diversification.

The orchard of *Gossypium* spp. maintained in Multan is a unique resource. Plants here are allowed to grow undisturbed and are re-grown from seeds only when they die. In many cases they represent genotypes that are present nowhere else in the country and the selection pressures they exerted on the viruses they harbor are thus likely to be entirely different to those in the widely grown *G. hirsutum* or in the annual weeds which may harbor the cotton viruses in the off season. Since the orchard has been maintained for some 40 years, it is likely that distinct virus populations have been maintained by insect transmission between plants within the orchard. There will of course have been introductions of viruses from the surrounding ecosystem but the numbers of bottlenecks, due to insect transmission and the death of plants, is likely to have been significantly less. The diversity of begomoviruses and associated components we have identified is wholly consistent with these assumptions. The analysis presented here thus provides an indication of the begomoviruses and their associated satellites that are, or have been, present in the environment, as well as an indication of their potential for evolution by component exchange and recombination. The study also highlights the effect of plant host background and, possibly, agricultural practices can have on plant virus populations. The plants in the orchard are under the same environmental pressures as the cotton plants in the adjacent farmer's fields, with the possible exception that no control measure for viruses or insects vectors (insecticides) are implemented in the orchard, and are inoculated with the same viruses carried by *B. tabaci* as the cotton plants in the farmer's fields, yet they contain an entirely different set of begomoviruses and associated satellites. At the time of sampling, only a single virus species, *Cotton leaf curl Burewala virus* [27], and its betasatellite (CLCuMuB^Bur^ [27], [28]) was present in the cultivated cotton surrounding the orchard. Cotton is by far the predominant crop in this region during the summer months when the insect vector, *B. tabaci*, is active.

The isolates of CLCuBuV characterized here differ from those identified earlier identified in having an intact TrAP gene. Amrao *et al.* (2010) showed that CLCuBuV, cloned for the most part from *G. hirsutum* plants carrying the CP-15/2-LRA-5166 derived resistance [20] to viruses of the Multan strain of CLCuD and showing severe CLCuD symptoms, lacked an intact TrAP [27]; a finding since confirmed for CLCuBuV originating from India [51], [52]. A hypothesis was put forward suggesting that the TrAP protein may be the avirulence determinant recognized by the resistance gene(s) of CP-15/2-LRA-5166 derived varieties, which could have selected for a virus lacking this gene. The close proximity of the orchard to the area where the resistance breaking virus, CLCuBuV, was first reported may suggest that CLCuBuV originated in the orchard. Certainly there is the distinct possibility that this, and other viruses, could originate from plants, such as the *Gossypium* spp. in the orchard, which harbor a great diversity of begomoviruses and associated components over a prolonged period – a situation that promotes recombination and components exchange.

Despite the preponderance of CLCuBuV in the environment surrounding the orchard at the time of sampling, the cotton species contained begomoviruses in addition to CLCuBuV. CLCuRaV was identified in two cotton species but has only recently been identified in Pakistan for the first time, infecting tomato in Faisalabad [53]. It has not been identified in cotton in Pakistan but has previously been identified in *G. hirsutum* affected by CLCuD in India [22].

The presence of a begomovirus DNA-B component in cotton is surprising. No DNA-B components have previously been identified in *G. hirsutum* in Pakistan where only betasatellite-requiring begomoviruses have been identified. It is also surprising to find that the DNA-B is closely related to the DNA-B components of ICMV, JCMV and SLCMV. Although several other bipartite begomoviruses occur in Pakistan, such as *Tomato leaf curl New Delhi virus* [54], *Mungbean yellow mosaic India virus* [55] and *Squash leaf curl China virus* [56], ICMV, JCMV and SLCMV have so far not been identified in Pakistan. The origin of the DNA-B component thus remains a mystery. SLCMV is believed to be a monopartite virus which has “captured” a DNA-B component from ICMV [36]. Thus SLCMV DNA-B has most of the sequence of the ICMV DNA-B, but contains the ori of SLCMV DNA-A; presumably obtained by recombination with SLCMV DNA-A – a process referred to as “origin donation” [36]. Closer analysis of the DNA-B components shows them to carry the *ori* of GPMLCuV. This indicates that origin donation has occurred which is supported by all the DNA-B molecules having the same predicted iteron sequences as GPMLCuV that differ from those of the ACMV DNA-A components isolated here. This is consistent with GPMLCuV being the cognate “DNA-A” of these DNA-B components. However, in three cotton species (*G. darwinii*, *G. stocksii* and *G. somalense*) no begomovirus (or DNA-A component) was identified that has iteron sequences compatible with the DNA-B components. The precise mechanism for maintenance of the DNA-B components in these cotton species thus remains unclear and will require further investigation.

ICMV, JCMV and SLCMV are not known to infect *Gossypium* spp. The DNA-B components of bipartite begomoviruses encode two products (NSP and MP) that mediate intra- and intercellular movement of viruses in hosts, requiring interaction with both the virus (specifically viral DNA) and host factors [57], [58], [59], [60], [61], [62]. The question thus arises whether the DNA-B component identified here is functional in cotton or is maintained as a non-functional “satellite-like” molecule which makes no contribution to the infectivity of the virus(es) with which it is associated. This will require experimental verification, specifically infectivity studies with clones in cotton, which at this time is not possible. However, maintenance of the NSP and MP coding sequences suggests that there is a purifying selection for their maintenance, which in turn is good evidence that the genes are functional and thus act as virus movement proteins in *Gossypium* spp. If this turns out to be the case, this would be the first bipartite begomovirus identified infecting cotton in the OW. To investigate whether GPMLCuV can trans-replicate DNA-B, clones were inoculated to *N. benthamiana*. The DNA-B was maintained in *N. bethamiana* and together the two components induced symptoms that were distinct from plants infected with only GPMLCuV. Additionally the viral DNA levels were elevated, indicating that GPMLCuV can trans-replicate the DNA-B and the DNA-B contributes to the virus infection (this likely indicating that the genes encoded by the DNA-B are functional). Similarly GPMLCuV was inoculated with the DNA-B and CLCuMuB to *N. benthamiana* plants and the satellite was maintained, inducing severe symptoms. This indicates that GPMLCuV can potentially act as either a monopartite satellite-associated virus or as a bipartite virus. However, whether this is also the case in *G. hirsutum* remains to be determined and is an important question.

The identification of a JCMV-derived DNA-B component in these cotton species may indicate that ICMV, JCMV, SLCMV or a closely related species, sharing a DNA-B component with them, may be present in Pakistan. Possibly one or more of these viruses remain to be identified in weed hosts. Weeds of the family *Euphorbiaceae* and *Fabaceae* are known to be one of the few alternative hosts/reservoirs of the African cassava-infecting begomoviruses [63], [64], thus possibly JCMV has yet to be identified in such weeds.

*G. arboreum* and G. *herbaceum* are native to Asia and Africa and were found not to contain any begomoviruses and their associated components, confirming earlier reports [19], [23]. This has been taken to indicate that these cotton species have had a long association with the viruses present in these regions and have evolved efficient resistance mechanisms against them. This is clearly not the case for the NW cotton species, which are highly susceptible and appear to be selecting for unique viruses and their associated components in this orchard, in comparison to the *G. hirsutum* crop. Nevertheless, there is some inherent capacity for resistance to the OW begomoviruses in the genetic make-up of NW cotton, as seen in the resistance to the CLCuMuV selected from *G. hirsutum* by conventional breeding during the epidemic of the 1990s [20].

In addition to the monopartite begomovirus species which have previously been shown to be associated with CLCuD many of the cotton species examined here also contained a begomovirus which has not been identified previously. In common with the majority of viruses this has a recombinant origin with most of the sequence derived from CLCuMuV. The name *Gossypium punctatum mild leaf curl virus* (GPMLCuV) is proposed for this new species. It seems likely that this begomovirus species evolved in the orchard. The lack of apparent spread of GPMLCuV out of the orchard may suggest that *G. hisutum* is resistant to it, or that the virus is not well adapted to *G. hirsutum*.

Six of the cotton species were shown to contain ACMV DNA-A. In addition, the sequence of DNA-A isolate [PK:Mul:Som:08], (FJ218487), isolated from *G. somalense*, contains a small (approximately 68 nt) fragment derived from ACMV. ACMV occurs across sub-Saharan Africa [65] but has not been reported from elsewhere, including Pakistan and India. The presence of ACMV is surprising since cassava is not grown in Pakistan. There are long-standing trade routes between North Africa and Pakistan and some of the peoples of coastal Pakistan have their ethnic roots in Sudan. Also there are large expatriate south Asian communities in many East African countries. These are all possible routes for transfer of viruses between regions. It has recently been suggested that *Bean yellow dwarf virus* (BeYDV), a dicot-infecting mastrevirus, was introduced into southern Africa by this means: BeYDV occurs in both southern Africa and in Pakistan [66].

ACMV is a bipartite virus and requires the presence of a DNA-B, which encodes movement functions, to infect plants [6]. However, in none of the plants was there any evidence for the presence of ACMV DNA-B. It is noticeable that all plants shown to contain ACMV DNA-A also contained the JCMV-like DNA-B. However, the predicted iterons of ACMV and those of the DNA-B components detected are distinct, suggesting that the ACMV Rep would be unable to trans-replicate these DNA-B components. However, this will need to be confirmed experimentally, as exceptions are possible. Many of the ACMV DNA-A clones isolated from the cotton species are defective. This may suggest that the ACMV DNA-A component is being maintained as a satellite-like molecule, with movement functions (to complement the missing DNA-B) and insect transmission (for those ACMV components lacking the CP) being provided *in trans* from another begomovirus.

The high levels of sequence identity between the ACMV DNA-A components identified in cotton and ACMV isolates originating from western Africa (particularly Cameroon and Ivory Coast) suggest that this is the geographical origin of the ACMV that has been introduced into Pakistan. This finding also suggests that the introduction has been quite recent; the virus having diverged little from the isolates present in Africa. In order to rule out any possibility of contamination during the cloning process, the orchard in Multan was re-sampled in 2008 and the presence of ACMV DNA-A was confirmed. The finding that some isolates of CLCuMuV and CLCuGeB contain small recombinant fragments of ACMV and CLCuMuB respectively, further strengthens the suggestion that there has been exchange of viruses between Africa and southern Asia.

Only a single species of betasatellite, the CLCuD-associated betasatellite CLCuMuB, was shown to be present in the diverse cotton species. CLCuMuB has been shown to be essential for inducing disease in *G. hirsutum* [19]. In the absence of this betasatellite CLCuMuV was shown to be very poorly infectious to *G. hirsutum* and to induce atypical symptoms [67]. Betasatellites encode a single gene product (βC1) that is a pathogenicity determinant which suppresses PTGS [15], [16]. Expression of the CLCuMuB βC1 from a PVX vector has been shown to induce all the symptoms typical of CLCuD [68]. It is thus no surprise to find CLCuMuB in these exotic cotton species, indicating that, for many of them, the begomoviruses they harbor likely also require the additional functions provided by this betasatellite to effectively infect them. However, three cotton species (*G. gossypioides*, *G. somalense* and *G. lobatum*) were identified which contain viruses that have previously been shown to be betasatellite-requiring but in which no betasatellites were detected. For example, in *G. somalense* the presence of CLCuRaV was shown but without a betasatellite. Additionally, for *G. mustelinum*, although the presence of the betasatellite-requiring CLCuKoV was shown, only CLCuMuB mutants lacking the βC1 gene were identified. These results suggest that, in contrast to *G. hirsutum*, in some instances infection of these cotton species does not require the presence of the functions provided by the CLCuMuB. Nevertheless, in most cases, the severe symptom phenotype correlated with the presence of CLCuMuB.

Although for *G. davidsonii* the presence of the betasatellite was shown, this species exhibited only mild symptoms, whereas *G. gossypioides* did not contain the betasatellite, but showed very severe symptoms. These results suggest that disease in some cotton species can be induced by begomoviruses without CLCuMuB. The complexity with regards to the presence of so many distinct ssDNA molecules makes it difficult to draw any definite conclusions and the suggestion that begomoviruses can infect and induce disease in these species without a betasatellite will need to be confirmed experimentally. There is no information in the literature on the susceptibility of these species to begomoviruses and no reason to believe that they should require a betasatellite for begomovirus infection. In the NW, from where many of these cotton species originate, no betasatellites have been identified and it is bipartite begomoviruses that commonly infect *G. hirsutum* [69].

The orchard in Multan was originally established as an aid to improving *G. hirsutum*, the major component of which has been efforts to identify exotic sources of resistance to CLCuD. It is evident, and consistent with earlier reports, that the A genome cotton species (such as *G. arboreum*) are immune to CLCuD. The fact that no varieties of these species have been shown to be susceptible might indicate that this is a non-host interaction. The remaining species, having D, E and AD genomes, are almost universally susceptible. The one exception here is *G. therburi* which showed no symptoms, nor the presence of any of the geminivirus components. For this species it might be worthwhile examining further varieties to determine whether the lack of support for begomovirus infection constitutes a non-host or a resistance type interaction. If it is a resistance interaction (thus *G. therburi* contains resistance genes for the viruses causing CLCuD) this might be a useful source of resistance genes for incorporation into *G. hirsutum*. If *G. therburi* is a non-host, the fact that all A×D hybrid species are susceptible, might indicate that this species is not useful for improving the CLCuD resistance of *G. hirsutum* since it appears that D genome susceptibility is dominant over A genome non-host resistance (suggesting that the A genome lacks some factor that is essential for infection by begomoviruses).

Since geminiviruses are not seed transmissible, it is unlikely that CGs were introduced with the introduction of cotton species from the NW. The close relationship of CGs to other OW begomoviruses (including their having a V2 gene, which is lacking in NW begomoviruses) is strong evidence that these are viruses native to the sub-Continent which have adapted to infect *G. hirsutum*. Some 149 years after the introduction of *G. hirsutum*, CLCuD was first noted in the vicinity of Multan [49]. TMRCA estimates indicate that for two major groups of CGs (CLCuMuV and CLCuKoV/CLCuRaV: Figure 7A) diversification started in the late 19^th^ century. The exact nature of the begomoviruse(s) and satellite(s) involved at the time of the first incidence is unknown. In our analysis for date estimates, it is clear that CLCuMuV was present in the early 20^th^ century. However, CLCuMuB seems to emerge after 1963, consistent with the time of the first emergence of CLCuD on *G. hirsutum*. It is also evident that, at the time of the first epidemic during earlier 1990s all the CGs species were present. However, the fact that CLCuMuV was the most widespread (based on the numbers of sequences isolated) prior to resistance breaking, that CLCuMuV appears to have donated sequences to numerous other CGs by recombination and that only CLCuMuB appears able to induce (in association with CGs) CLCuD, leads to the not unreasonable assumption that the first infection of cotton in the 1980s, that ultimately led to the epidemic, involved CLCuMuV and CLCuMuB. The introduction of resistant cotton varieties in the 1990s was a stringent bottleneck through which only one virus was able to pass. It remains unclear what the molecular basis for resistance breaking is. Both CLCuBuV and CLCuMuB^Bur^ show changes with respect to the viruses and betasatellite occurring pre-resistance breaking. Unfortunately the lack of an infectivity system for cotton prevents a more detailed investigation at this time. However, what happened in 2001 is an indication of the apparent ease with which conventional resistance can be broken and does not bode well for future conventional host-plant resistance, which may be identified

Four distinct alphasatellites (CLCuMuA, CLCuShA, GDarSLA and GMusSLA) have been shown to be associated with CLCuD in Pakistan. The TMCRA estimates suggest that GMusSLA was present before the disease appeared in an epidemic form. However, GDarSLA seems to have evolved more recently, in the early 90s. The role of alphasatellites in begomovirus-betasatellite complexes remains unclear. Several of the *Gossypium* species studied here contain an unusual diversity of satellites and exhibit mild symptoms. GDarSLA, GMusSLA and AYVSGA have the capacity to ameliorate begomovirus symptoms in plants [47], [70]. It is thus possible that, in the *Gossypium* spp., this diversity of alphasatellites acts to reduce the pathogenicity of the begomoviruses/betasatellite and could thus promote virus diversification by allowing plants to tolerate infection. This together with the suggestion, from nucleotide substitution rates, that virus and satellites are under different evolutionary pressures and the finding that each *Gossypium* species is selecting for distinct satellites (a possible indication of little exchange of components between *Gossypium* spp.) may be the reasons for the accumulation of such a diversity of viruses and satellites in the orchard.

It is evident that the long term maintenance of the plants in Multan has led to a build-up of a diverse array of begomoviruses and begomovirus-associated satellites and has, more than likely, provided a crucible for the evolution of new species, strains and variants by mutation, recombination and component exchange. It is likely that the diversity identified in the plants in this orchard is a reflection of the diversity of begomoviruses and begomovirus-associated satellites that have been, or are, present in the environment. If this is the case, it is evident that the diversity is much greater than previously estimated by analyzing single isolates from single cultivated host plants. Likely this identification of a wider diversity results not only from the unusual nature of the plants in the orchard (exotic germplasm that has been maintained vegetatively over a very long period of time) but also from the ability of RCA to amplify circular DNA components without prior knowledge of the sequence to be amplified (a requirement that is a definite draw-back of the widely used PCR technique to clone geminivirus molecules). It is possibly coincidental that we have identified CLCuBuV with an intact TrAP gene in the orchard, whereas across the rest of the country it appears that CLCuBuV lacks this gene, possibly suggesting that this virus species could have originated in the wild species. However, there are other possible sources for this virus such as malvaceous perennials that are maintained long term in an infected state. A pertinent example here is *Hibiscus rosa-sinensis. Hibiscus* is a malvaceous perennial that is grown widely as an ornamental and hedge plant across Pakistan and India and, in areas where the environmental conditions are suitable (thus the same areas where cotton is grown), almost universally exhibits symptoms typical of CLCuD and harbors the components that cause CLCuD [26]). Such plants could thus, like the exotic *Gossypium* plants in Multan, be the source of new virus/satellite diversity. The identification, in our study, of a CLCuMuV isolate that contains some sequence derived from ACMV in *G. hirsutum* that did not harbor ACMV, is clear evidence that such new variants are spreading out of the orchard into the crop.

The take-home message from these finding is that this long term maintenance of such virus-infected perennials is unwise, particularly in areas where susceptible crops are grown. The advice must therefore be for the *Gossypium* orchard in Multan, as well as similar plant collections across Pakistan and India, to be removed each year and replanted from seed, since geminiviruses are not seed transmissible. For farmers, the removal of infected perennials, such as *Hibiscus* spp., would be a wise precaution in an effort to reduce the availability of overwintering hosts and potential sources of virus diversity. Farmers are already advised to remove cotton plants at the end of every season and not allow ratooning (regrowth) from the previous year's plants, in an effort to avoid build-up of virus early in the season. Although, with the benefit of hindsight, we can now say that maintaining the *Gossypium* orchard in the middle of the cotton growing belt of Pakistan was/is unwise, it has proven a valuable source of information on the diversity, evolution and adaptation of begomoviruses and their associated satellites.

## Materials and Methods

### Sample collection and DNA extraction

Fresh young leaves were frozen in liquid nitrogen and total genomic DNA was extracted by the CTAB method [71]. All necessary permits were obtained from USDA/APHIS and USDA/BRS to import the material to the Danforth Center.

### Amplification, cloning and sequencing of begomoviruses and their satellites

DNA was quantified using an Ultrospec3000 (Pharmacia Biotech) spectrophotometer and each DNA sample was diluted to 10 ng/µl. RCA reactions were prepared as recommended by manufacturer (TempliPhi™, GE Healthcare). Briefly, DNA (∼20 ng) was diluted in 5 µl of reaction buffer and denatured for 5 min at 95°C. After cooling at room temperature, 5 µl of sample buffer was added followed by 0.2 µl of φ29 DNA polymerase enzyme mix. The reaction was allowed to proceed for 16 hrs at 28°C and then stopped by heating at 65°C for 10 min. Approximately 1 µg of RCA product was digested with selected restriction endonucleases and bands of approx. 2800 nt and/or 1400 nt were selected for cloning. Selected restriction products were gel isolated and cloned in the plasmid vector pUC19 [72]. Due to the non-specific nature of the amplification of all circular DNA molecules by φ29 DNA polymerase, clones of betasatellites and alphasatellites were difficult to distinguish based only upon size. Colony hybridization [73] was performed to identify recombinant *E. coli* colonies harboring betasatellites using the βC1 gene of CLCuMuB (EU384588) as a probe. Cloned products were sequenced using a model 377 Automated DNA Sequencer (Perkin Elmer). All clones were sequenced, using the primer walking strategy, in both orientations with no ambiguities remaining.

### Sequence assembly, manipulation and analysis

Sequences were assembled and analyzed using the Lasergene (v.8) package (DNASTAR, Madison, Wisconsin). The sequences obtained were compared with sequences available in public databases using BLASTn (NCBI). The coding regions and coding capacities of virus/satellites clones were predicted using the EditSeq module of Lasergene.

### Phylogenetic, sequence distance analysis and detection of recombination

Sequences were aligned using Mega5 by applying the Clustal-W algorithm and resulting alignments were used for pairwise comparisons and construction of phylogenetic trees using the neighbor-joining algorithm [74]. Recombination was detected using RDP3 [75] available at http://darwin.uvigo.es/rdp/rdp.html. The default X-over panel was used for recombination analysis in all cases.

### Estimation for times of divergence

Divergence times for virus and satellite components associated with CLCuD were estimatedby Bayesian Marcov Chain Monte Carlo (MCMC), applied as implemented in BEAST v1.6 [76]. No out-group sequences were used in the BEAST analysis and the relationship based on tree topologies in the preliminary analysis (described earlier) was enforced as prior assumption for the Bayesian analysis. The year wise dates for each sequence were manually added in the tree dates panel of BEAST for TMRCA. The relaxed molecular clock (uncorrelated lognormal) along with exponential population growth models was set for further analysis as described previously for EACMV, TYLCV and influenza virus [50], [77], [78], [79]. Each species of begomovirus and satellite was divided into monophyletic taxa in the taxon-set tab of BEAUti module of BEAST. This was performed to ensure that during the dating estimates each species was kept under a monophyletic group. To ensure an adequate sample size, a chain length of 40,000,000 was used in the MCMC panel. The log and tree files generated by BEAST were combined in the LogCombinar (v.1.6.1) application of BEAST. The LogCombined trees were annotated using TreeAnnotator (v.1.6.1). MCC phylogenetic trees were calculated with 10–15% burn-in followed by visual inspection in TRACER (v.1.5; http://tree.bio.ed.ac.uk/software/tracer/). Phylogenetic trees were analyzed using FigTree (v.1.3.1; http://tree.bio.ed.ac.uk/software/figtree). Since sequence data produced prior to 2010 was used, this year was used as the offset for the time limit in the time scale panel of the FigTree package. The Log and Tree files of the analysis can be obtained from the authors upon request.

### Estimation of nucleotide substitution rates by Bayesian Skyline Plot

For all three datasets (CP, βC1 and alpha-Rep) nexus files were generated after aligning sequences in Mega5 as described above. The general time reverse (GTR+E) substitution model was chosen and the nucleotides dataset was partitioned into 3 sets (codon positions 1, 2 and 3). Coalescent Bayesian Skyline was chosen in the tree panel for the tree prior and each dataset was run for a chain length of 4×10^7^ to ensure an adequate sample size in the MCMC panel of the BEAUTi module in BEAST.

### Inoculation of plants

GPMLCuV (EU365617), DNA-B (EU384577), CLCuMuBMul (FJ607041) and CLCuMuB^Bur^ (EU384587) were introduced into *N. benthamiana* plants by biolistic inoculation. Briefly, the components were excised from their cloning vectors by restriction digestion and then circularized by ligation. Each component was then amplified by RCA, as described earlier [12]. Approximately, 100 ng of multimeric RCA product was coated on 1 µM gold particles and inoculated to *N. benthamiana* plants using a Helios gene gun (BioRad) as described previously [80]. For co-inoculation of components equal amounts of each component were coated on a single aliquot of gold particles. After inoculation, plants were maintained in a glasshouse and scored for infection at 2 week post-inoculation [81].

### Southern blot hybridization

Total DNA was extracted from inoculated plants at 15 dpi using a DNAeasy mini kit (Qiagen). Total DNA (∼500 ng) was resolved on 1.5% agarose gels and transferred to nylon membranes (Hybond N+, Amersham). Digoxygenin (DIG) labeled probes for each component were prepared using a PCR DIG Probe Synthesis Kit (Roche). For GPMLCuV primers for amplification of the C4 gene (5′-TCAGGGCCTCTGCTGCTGCATCATT-3′ and 5′-ATGGGTCTCTGCATATCCACGC-3′) and for the DNA-B component primers for the BV1 gene (5′-ATGAGAAATGTTGGTTATACTCTCC-3′ and 5′-TTAACCAATATATCGCATTACATA-3′) were used to PCR-amplify the DIG labeled probes. For betasatellites, the βC1 probe described earlier was used [82].

## Supporting Information

Figure S1 **Mutations of the virion-sense genes of ACMV DNA-A components isolated from cotton spp.** Alignment of the sequence of an isolate of ACMV (ACMV-[CM:Mg:98].AY211884) originating from Africa (Cameroon) with one of the ACMV isolates obtained from cotton spp. (ACMV-[PK:Mul:Dar:06].GQ169505). Conserved nucleotides are highlighted in red. The positions of the AV2 (purple box) and AV1 (CP; orange box) genes of ACMV-[CM:Mg:98] are shown. Gaps (−) were introduced into the sequences to optimise the alignment.
(TIF)

Figure S2 **Duplicated stem-loop sequences in the DNA-B components isolated from** ***Gossypium*** **species.** Alignment of the 3′ sequence, from the hairpin-loop structures (coordinate 1) onwards, of the DNA-B components isolated in this study. Duplicated sequences spanning the right (3′) leg of the stem loop structure (underlined) are highlighted in red. The 3′ repeat is the *bona fide* right (3′) leg of the components. The blue and orange boxes highlight sequences originating from BYVMV and CLCuMuV, respectively.
(TIF)

Figure S3 **Phylogenetic analysis of betasatellite sequences.** This tree differs from that in Figure 5 only in containing more sequences; sequences representing all so far identified betasatellite species. For other details about the tree see the figure legend of Figure 5.
(TIF)

Table S1 **List of virus genomes and genomic components isolated from** ***Gossypium*** **species.**
(DOC)

Table S2 **ACMV DNA-A clones isolated from cotton species.**
(DOC)

Table S3 **Details of recombination between CGs detected using RDP3.**
(DOC)

Table S4 **CLCuMuB clones isolated from** ***Gossypium*** **species.**
(DOC)

Table S5 **Alphasatellites isolated from** ***Gossypium*** **species.**
(DOC)

Table S6 **Upper and lower bounds of the 95% highest posterior density (HPD) estimates for divergence dates of CLCuMuV-encoded CP, CLCuMuB-encoded βC1 and GDarSLA-encoded Rep.**
(DOC)

## References

[1] Fauquet C, Briddon R, Brown J, Moriones E, Stanley J, et al (2008) Geminivirus strain demarcation and nomenclature. Arch Virol 153: 783–821. 18256781 10.1007/s00705-008-0037-6

[2] Brown JK, Fauquet CM, Briddon RW, Zerbini M, Moriones E, et al.. (2011) Geminiviridae. In: King AMQ, Adams MJ, Carstens EB, Lefkowitz EJ, eds. Virus Taxonomy - Ninth Report of the International Committee on Taxonomy of Viruses. London, Waltham, San Diego: Associated Press, Elsevier Inc. pp. 351–373.

[3] Varma A, Malathi VG (2003) Emerging geminivirus problems: A serious threat to crop production. Ann Appl Biol 142: 145–164.

[4] Moffat AS (1999) Geminiviruses emerge as serious crop threat. Science 286: 1835.

[5] Briddon RW, Stanley J (2006) Subviral agents associated with plant single-stranded DNA viruses. Virology 344: 198–210. 16364750 10.1016/j.virol.2005.09.042

[6] Stanley J (1983) Infectivity of the cloned geminivirus genome requires sequences from both DNAs. Nature 305: 643–645.

[7] Rojas MR, Hagen C, Lucas WJ, Gilbertson RL (2005) Exploiting chinks in the plant's armor: Evolution and emergence of geminiviruses. Ann Rev Phytopathol 43: 361–394. 16078889 10.1146/annurev.phyto.43.040204.135939

[8] Stanley J, Gay MR (1983) Nucleotide sequence of cassava latent virus DNA. Nature 301: 260–262.

[9] Argüello-Astorga GR, Ruiz-Medrano R (2001) An iteron-related domain is associated to Motif 1 in the replication proteins of geminiviruses: identification of potential interacting amino acid-base pairs by a comparative approach. Arch Virol 146: 1465–1485. 11676411 10.1007/s007050170072

[10] Chatterji A, Chatterji U, Beachy RN, Fauquet CM (2000) Sequence parameters that determine specificity of binding of the replication-associated protein to its cognate site in two strains of tomato leaf curl virus-New Delhi. Virology 273: 341–350. 10915605 10.1006/viro.2000.0434

[11] Li Z, Xie Y, Zhou X (2005) *Tobacco curly shoot virus* DNA β is not necessary for infection but intensifies symptoms in a host-dependent manner. Phytopathology 95: 902–908. 18944412 10.1094/PHYTO-95-0902

[12] Nawaz-ul-Rehman MS, Mansoor S, Briddon RW, Fauquet CM (2009) Maintenance of an Old World betasatellite by a New World helper begomovirus and possible rapid adaptation of the betasatellite. J Virol 83: 9347–9355. 19570867 10.1128/JVI.00795-09 PMC2738271

[13] Saunders K, Briddon RW, Stanley J (2008) Replication promiscuity of DNA-β satellites associated with monopartite begomoviruses; deletion mutagenesis of the ageratum yellow vein virus DNA-β satellite localizes sequences involved in replication. J Gen Virol 89: 3165–3172. 19008407 10.1099/vir.0.2008/003848-0

[14] Saeed M, Zafar Y, Randles JW, Rezaian MA (2007) A monopartite begomovirus-associated DNA β satellite substitutes for the DNA B of a bipartite begomovirus to permit systemic infection. J Gen Virol 88: 2881–2889. 17872543 10.1099/vir.0.83049-0

[15] Cui X, Li G, Wang D, Hu D, Zhou X (2005) A begomovirus DNAβ-encoded protein binds DNA, functions as a suppressor of RNA silencing, and targets the cell nucleus. J Virol 79: 10764–10775. 16051868 10.1128/JVI.79.16.10764-10775.2005 PMC1182626

[16] Saunders K, Norman A, Gucciardo S, Stanley J (2004) The DNA β satellite component associated with ageratum yellow vein disease encodes an essential pathogenicity protein (βC1). Virology 324: 37–47. 15183051 10.1016/j.virol.2004.03.018

[17] Briddon RW, Bull SE, Amin I, Mansoor S, Bedford ID, et al (2004) Diversity of DNA 1: a satellite-like molecule associated with monopartite begomovirus-DNA β complexes. Virology 324: 462–474. 15207631 10.1016/j.virol.2004.03.041

[18] Moulherat C, Tengberg M, Haquet J-F, Mille B (2002) First Evidence of cotton at Neolithic Mehrgarh, Pakistan: Analysis of mineralized fibres from a copper bead. J Archaeol Sci 29: 1393–1401.

[19] Briddon RW, Mansoor S, Bedford ID, Pinner MS, Saunders K, et al (2001) Identification of DNA components required for induction of cotton leaf curl disease. Virology 285: 234–243. 11437658 10.1006/viro.2001.0949

[20] Rahman M, Hussain D, Malik TA, Zafar Y (2005) Genetics of resistance to cotton leaf curl disease in *Gossypium hirsutum* . Plant Pathol 54: 764–772.

[21] Mansoor S, Amin I, Iram S, Hussain M, Zafar Y, et al (2003) Breakdown of resistance in cotton to cotton leaf curl disease in Pakistan. Plant Pathol 52: 784–784.

[22] Kirthi N, Priyadarshini CGP, Sharma P, Maiya SP, Hemalatha V, et al (2004) Genetic variability of begomoviruses associated with cotton leaf curl disease originating from India. Arch Virol 149: 2047–2057. 15669112 10.1007/s00705-004-0352-5

[23] Mansoor S, Briddon RW, Bull SE, Bedford ID, Bashir A, et al (2003) Cotton leaf curl disease is associated with multiple monopartite begomoviruses supported by single DNA β. Arch Virol 148: 1969–1986. 14551819 10.1007/s00705-003-0149-y

[24] Zhou X, Liu Y, Robinson D, Harrison B (1998) Four DNA-A variants among Pakistani isolates of cotton leaf curl virus and their affinities to DNA-A of geminivirus isolates from okra. J Gen Virol 79: 915–923. 9568988 10.1099/0022-1317-79-4-915

[25] Tahir MN, Amin I, Briddon RW, Mansoor S (2011) The merging of two dynasties-identification of an african cotton leaf curl disease-associated begomovirus with cotton in Pakistan. PLoS ONE 6: e20366. 21637815 10.1371/journal.pone.0020366 PMC3102712

[26] Briddon RW, Bull SE, Amin I, Idris AM, Mansoor S, et al (2003) Diversity of DNA β, a satellite molecule associated with some monopartite begomoviruses. Virology 312: 106–121. 12890625 10.1016/s0042-6822(03)00200-9

[27] Amrao L, Amin I, Shahid MS, Briddon RW, Mansoor S (2010) Cotton leaf curl disease in resistant cotton is associated with a single begomovirus that lacks an intact transcriptional activator protein. Virus Res 152: 153–163. 20600387 10.1016/j.virusres.2010.06.019

[28] Amin I, Mansoor S, Amrao L, Hussain M, Irum S, et al (2006) Mobilisation into cotton and spread of a recombinant cotton leaf curl disease satellite. Arch Virol 151: 2055–2065. 16732497 10.1007/s00705-006-0773-4

[29] Wendel JF, Brubaker C, Alvarez I, Cronn R, Stewart JM (2009) Evolution and natural history of the cotton genus. Genet Genom Cotton: Springer New York. pp. 1–20.

[30] Akhtar KP, Haidar S, Khan MKR, Ahmad M, Sarwar N, et al (2010) Evaluation of Gossypium species for resistance to cotton leaf curl Burewala virus. Ann Appl Biol 157: 135–147.

[31] Homs M, Kober S, Kepp G, Jeske H (2008) Mitochondrial plasmids of sugar beet amplified via rolling circle method detected during curtovirus screening. Virus Res 136: 124–129. 18562034 10.1016/j.virusres.2008.04.027

[32] Fauquet CM, Bisaro DM, Briddon RW, Brown JK, Harrison BD, et al (2003) Revision of taxonomic criteria for species demarcation in the family *Geminiviridae*, and an updated list of begomovirus species. Arch Virol 148: 405–421. 12557003 10.1007/s00705-002-0957-5

[33] Lefeuvre P, Lett J-M, Varsani A, Martin DP (2009) Widely conserved recombination patterns amongst single stranded DNA viruses. J Virol 83: 2697–2707. 19116260 10.1128/JVI.02152-08 PMC2648288

[34] Lefeuvre P, Martin DP, Hoareau M, Naze F, Delatte H, et al (2007) Begomovirus ‘melting pot’ in the south-west Indian Ocean islands: molecular diversity and evolution through recombination. J Gen Virol 88: 3458–3468. 18024917 10.1099/vir.0.83252-0

[35] Gao S, Qu J, Chua N-H, Ye J (2010) A new strain of *Indian cassava mosaic virus* causes a mosaic disease in the biodiesel crop *Jatropha curcas* . Arch Virol 155: 607–612. 20224893 10.1007/s00705-010-0625-0

[36] Saunders K, Salim N, Mali VR, Malathi VG, Briddon R, et al (2002) Characterisation of Sri Lankan cassava mosaic virus and Indian cassava mosaic virus: Evidence for acquisition of a DNA B component by a monopartite begomovirus. Virology 293: 63–74. 11853400 10.1006/viro.2001.1251

[37] Briddon R, Brown J, Moriones E, Stanley J, Zerbini M, et al (2008) Recommendations for the classification and nomenclature of the DNA-β satellites of begomoviruses. Arch Virol 153: 763–781. 18247103 10.1007/s00705-007-0013-6

[38] Tao X, Zhou X (2008) Pathogenicity of a naturally occurring recombinant DNA satellite associated with tomato yellow leaf curl China virus. J Gen Virol 89: 306–311. 18089755 10.1099/vir.0.83388-0

[39] Saunders K, Bedford ID, Stanley J (2001) Pathogenicity of a natural recombinant associated with ageratum yellow vein disease: Implications for geminivirus evolution and disease aetiology. Virology 282: 38–47. 11259188 10.1006/viro.2000.0832

[40] Das S, Roy A, Ghosh R, Paul S, Acharyya S, et al (2008) Sequence variability and phylogenetic relationship of betasatellite isolates associated with yellow vein mosaic disease of mesta in India. Virus Genes 37: 414–424. 18807163 10.1007/s11262-008-0287-0

[41] Chatterjee A, Ghosh S (2007) Association of a satellite DNA β molecule with mesta yellow vein mosaic disease. Virus Genes 35: 835–844. 17763932 10.1007/s11262-007-0160-6

[42] Paul S, Ghosh R, Roy A, Mir JI, Ghosh SK (2006) Occurrence of a DNA β-containing begomovirus associated with leaf curl disease of kenaf (*Hibiscus cannabinus* L.) in India. Aust Plant Dis Notes 1: 29–30.

[43] Tahir MN, Amin I, Briddon RW, Mansoor S (2011) The merging of two dynasties–identification of an African cotton leaf curl disease-associated begomovirus with cotton in Pakistan. PLoS ONE 6: e20366. 21637815 10.1371/journal.pone.0020366 PMC3102712

[44] Amrao L, Akhter S, Tahir MN, Amin I, Briddon RW, et al (2010) Cotton leaf curl disease in Sindh province of Pakistan is associated with recombinant begomovirus components. Virus Res 153: 161–165. 20621137 10.1016/j.virusres.2010.07.003

[45] Mansoor S, Khan SH, Bashir A, Saeed M, Zafar Y, et al (1999) Identification of a novel circular single-stranded DNA associated with cotton leaf curl disease in Pakistan. Virology 259: 190–199. 10364503 10.1006/viro.1999.9766

[46] Saunders K, Bedford ID, Stanley J (2002) Adaptation from whitefly to leafhopper transmission of an autonomously replicating nanovirus-like DNA component associated with ageratum yellow vein disease. J Gen Virol 83: 907–913. 11907341 10.1099/0022-1317-83-4-907

[47] Idris AM, Shahid MS, Briddon RW, Khan AJ, Zhu JK, et al (2011) An unusual alphasatellite associated with monopartite begomoviruses attenuates symptoms and reduces betasatellite accumulation. J Gen Virol 92: 706–717. 21084498 10.1099/vir.0.025288-0

[48] Paprotka T, Metzler V, Jeske H (2010) The first DNA 1-like α satellites in association with New World begomoviruses in natural infections. Virology 404: 148–157. 20553707 10.1016/j.virol.2010.05.003

[49] Hussain T, Tahir M, Mahmood T (1991) Cotton leaf curl virus. Pak J Phytopathol 3: 57–61.

[50] Duffy S, Holmes EC (2009) Validation of high rates of nucleotide substitution in geminiviruses: phylogenetic evidence from East African cassava mosaic viruses. J Gen Virol 90: 1539–1547. 19264617 10.1099/vir.0.009266-0 PMC4091138

[51] Zaffalon V, Mukherjee S, Reddy V, Thompson J, Tepfer M (2012) A survey of geminiviruses and associated satellite DNAs in the cotton-growing areas of northwestern India. Arch Virol 157: 483–495. 22209785 10.1007/s00705-011-1201-y

[52] Rajagopalan P, Naik A, Katturi P, Kurulekar M, Kankanallu R, et al (2012) Dominance of resistance-breaking cotton leaf curl Burewala virus (CLCuBuV) in northwestern India. Arch Virol 157: 855–868. 22307170 10.1007/s00705-012-1225-y

[53] Shahid MS, Mansoor S, Briddon RW (2007) Complete nucleotide sequences of cotton leaf curl Rajasthan virus and its associated DNA β molecule infecting tomato. Arch Virol 152: 2131–2134. 17703290 10.1007/s00705-007-1043-9

[54] Padidam M, Beachy RN, Fauquet CM (1995) Tomato leaf curl geminivirus from India has a bipartite genome and coat protein is not essential for infectivity. J Gen Virol 76: 25–35. 7844539 10.1099/0022-1317-76-1-25

[55] Ilyas M, Qazi J, Mansoor S, Briddon RW (2010) Genetic diversity and phylogeography of begomoviruses infecting legumes in Pakistan. J Gen Virol 91: 2091–2101. 20375225 10.1099/vir.0.020404-0

[56] Tahir M, Haider MS, Briddon RW (2010) First report of Squash leaf curl China virus in Pakistan. Aust Plant Dis Notes 5: 21–24.

[57] Lewis JD, Lazarowitz SG (2010) *Arabidopsis* synaptotagmin SYTA regulates endocytosis and virus movement protein cell-to-cell transport. Proc Natl Acad Sci USA 107: 2491–2496. 20133785 10.1073/pnas.0909080107 PMC2823903

[58] Carvalho MF, Turgeon R, Lazarowitz SG (2006) The geminivirus nuclear shuttle protein NSP inhibits the activity of AtNSI, a vascular-expressed *Arabidopsis* acetyltransferase regulated with the sink-to-source transition. Plant Physiol 140: 1317–1330. 16461385 10.1104/pp.105.075556 PMC1435821

[59] Carvalho MF, Lazarowitz SG (2004) Interaction of the movement protein NSP and the *Arabidopsis* acetyltransferase AtNSI is necessary for cabbage leaf curl geminivirus infection and pathogenicity. J Virol 78: 11161–11171. 15452236 10.1128/JVI.78.20.11161-11171.2004 PMC521842

[60] McGarry RC, Barron YD, Carvalho MF, Hill JE, Gold D, et al (2003) A novel *Arabidopsis* acetyltransferase interacts with the geminivirus movement protein NSP. Plant Cell 15: 1605–1618. 12837950 10.1105/tpc.012120 PMC165404

[61] Gilbertson RL, Sudarshana M, Jiang H, Rojas MR, Lucas WJ (2003) Limitations on geminivirus genome size imposed by plasmodesmata and virus-encoded movement protein: Insights into DNA trafficking. Plant Cell 15: 2578–2591. 14555695 10.1105/tpc.015057 PMC280562

[62] Rojas MR, Noueiry AO, Lucas WJ, Gilbertson RL (1998) Bean dwarf mosaic geminivirus movement proteins recognize DNA in a form- and size-specific manner. Cell 95: 105–113. 9778251 10.1016/s0092-8674(00)81786-9

[63] Alabi O, Ogbe F, Bandyopadhyay R, Lava Kumar P, Dixon A, et al (2008) Alternate hosts of African cassava mosaic virus and East African cassava mosaic Cameroon virus in Nigeria. Arch Virol 153: 1743–1747. 18661095 10.1007/s00705-008-0169-8

[64] Monde G, Walangululu J, Winter S, Bragard C (2010) Dual infection by cassava begomoviruses in two leguminous species (*Fabaceae*) in Yangambi, Northeastern Democratic Republic of Congo. Arch Virol 155: 1865–1869. 20680361 10.1007/s00705-010-0772-3

[65] Patil BL, Fauquet CM (2009) Cassava mosaic geminiviruses: actual knowledge and perspectives. Mol Plant Pathol 10: 685–701. 19694957 10.1111/j.1364-3703.2009.00559.x PMC6640248

[66] Nahid N, Amin I, Mansoor S, Rybicki E, van der Walt E, et al (2008) Two dicot-infecting mastreviruses (family; *Geminiviridae*) occur in Pakistan. Arch Virol 153: 1441–1451. 18566736 10.1007/s00705-008-0133-7

[67] Briddon RW, Mansoor S, Bedford ID, Pinner MS, Markham PG (2000) Clones of cotton leaf curl geminivirus induce symptoms atypical of cotton leaf curl disease. Virus Genes 20: 19–26. 10766303 10.1023/a:1008151921937

[68] Qazi J, Amin I, Mansoor S, Iqbal MJ, Briddon RW (2007) Contribution of the satellite encoded gene βC1 to cotton leaf curl disease symptoms. Virus Res 128: 135–139. 17482706 10.1016/j.virusres.2007.04.002

[69] Idris AM, Brown JK (2004) *Cotton leaf crumple virus* is a distinct western hemisphere begomovirus species with complex evolutionary relationships indicative of recombination and reassortment. Phytopathology 94: 1068–1074. 18943795 10.1094/PHYTO.2004.94.10.1068

[70] Nawaz-Ul-Rehman MS, Nahid N, Mansoor S, Briddon RW, Fauquet CM (2010) Post-transcriptional gene silencing suppressor activity of two non-pathogenic alphasatellites associated with a begomovirus. Virology 405: 300–308. 20598726 10.1016/j.virol.2010.06.024

[71] Doyle JJ, Doyle JL (1987) A rapid DNA isolation procedure for small quantities of fresh leaf tissue. Phytochem Bull 19: 11–15.

[72] Yanisch-Perron C, Vieira J, Messing J (1985) Improved M13 phage cloning vectors and host strains: nucleotide sequences of the M13mpl8 and pUC19 vectors. Gene 33: 103–119. 2985470 10.1016/0378-1119(85)90120-9

[73] Cami B, Kourilsky P (1978) Screening of cloned recombinant DNA in bacteria by *in situ* colony hybridization. Nucleic Acids Res 5: 2381–2390. 353741 10.1093/nar/5.7.2381 PMC342171

[74] Tamura K, Peterson D, Peterson N, Stecher G, Nei M, et al (2011) MEGA5: Molecular evolutionary genetics analysis using maximum likelihood, evolutionary distance, and maximum parsimony methods. Mol Bio Evol 10.1093/molbev/msr121 PMC3203626 21546353

[75] Martin DP, Lemey P, Lott M, Moulton V, Posada D, et al (2010) RDP3: a flexible and fast computer program for analyzing recombination. Bioinformatics 26: 2462–2463. 20798170 10.1093/bioinformatics/btq467 PMC2944210

[76] Drummond A, Rambaut A (2007) BEAST: Bayesian evolutionary analysis by sampling trees. BMC Evol Biol 7: 214. 17996036 10.1186/1471-2148-7-214 PMC2247476

[77] Bataille A, van der Meer F, Stegeman A, Koch G (2011) Evolutionary analysis of inter-farm transmission dynamics in a highly pathogenic avian influenza epidemic. PLoS Pathog 7: e1002094. 21731491 10.1371/journal.ppat.1002094 PMC3121798

[78] Lefeuvre P, Martin DP, Harkins G, Lemey P, Gray AJA, et al (2010) The spread of tomato yellow leaf curl virus from the middle East to the world. PLoS Pathog 6: e1001164. 21060815 10.1371/journal.ppat.1001164 PMC2965765

[79] Duffy S, Holmes EC (2008) Phylogenetic evidence for rapid rates of molecular evolution in the single-stranded DNA begomovirus Tomato yellow leaf curl virus (TYLCV). J Virol 82: 957–965. 17977971 10.1128/JVI.01929-07 PMC2224568

[80] Ariyo OA, Atiri GI, Dixon AGO, Winter S (2006) The use of biolistic inoculation of cassava mosaic begomoviruses in screening cassava for resistance to cassava mosaic disease. J Virol Methods 137: 43–50. 16839615 10.1016/j.jviromet.2006.05.031

[81] Fauquet C, Fargette D (1990) African cassava mosaic virus: etiology, epidemiology and control. Plant Dis 74: 404–411.

[82] Nawaz-ul-Rehman MS, Nahid N, Mansoor S, Briddon RW, Fauquet CM (2010) Post-transcriptional gene silencing suppressor activity of two non-pathogenic alphasatellites associated with a begomovirus. Virology 405: 300–308. 20598726 10.1016/j.virol.2010.06.024

