# Supplementary figures and images for: A Melting Pot of Old World Begomoviruses and Their Satellites Infecting a Collection of Gossypium Species in Pakistan
Source: PLoS One. 2012 Aug 10;7(8):e40050. doi: 10.1371/journal.pone.0040050 (PMC3416816; doi:10.1371/journal.pone.0040050)

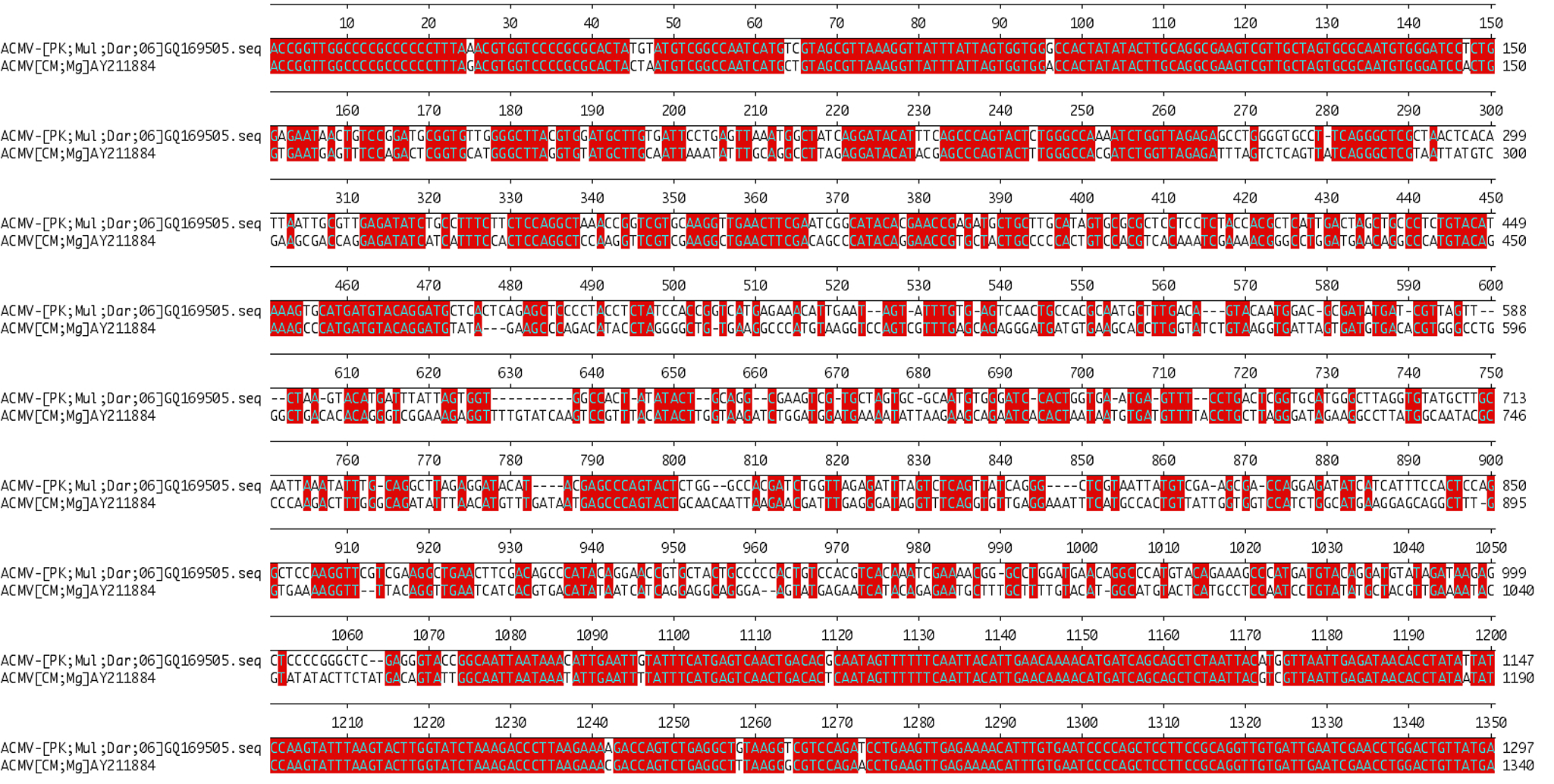

Supplement: Figure S1 — Mutations of the virion-sense genes of ACMV DNA-A components isolated from cotton spp. Alignment of the sequence of an isolate of ACMV (ACMV-[CM:Mg:98].AY211884) originating from Africa (Cameroon) with one of the ACMV isolates obtained from cotton spp. (ACMV-[PK:Mul:Dar:06].GQ169505). Conserved nucleotides are highlighted in red. The positions of the AV2 (purple box) and AV1 (CP; orange box) genes of ACMV-[CM:Mg:98] are shown. Gaps (−) were introduced into the sequences to optimise the alignment. (TIF) [file pone.0040050.s001.tif]

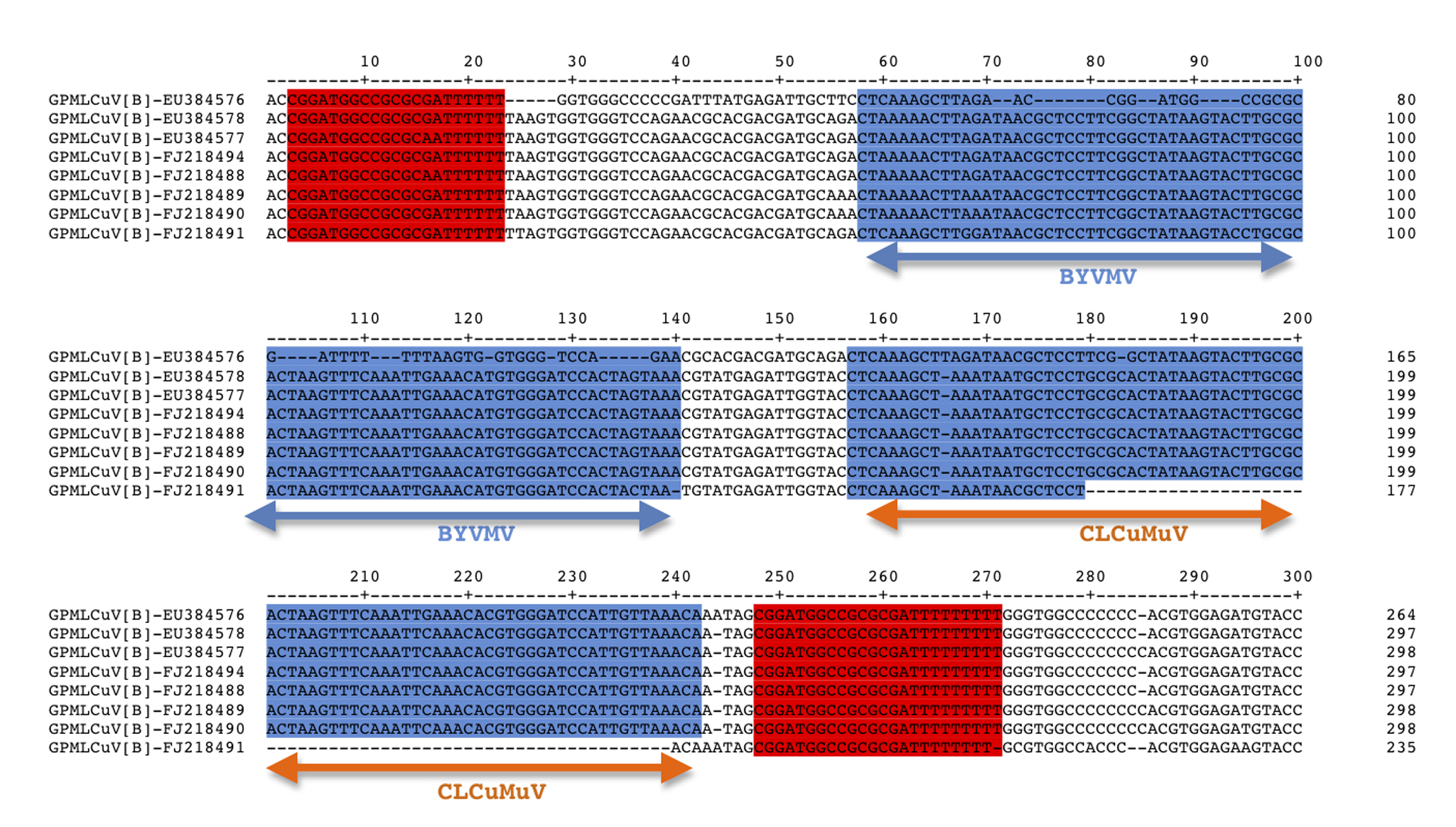

Supplement: Figure S2 — Duplicated stem-loop sequences in the DNA-B components isolated from Gossypium species. Alignment of the 3′ sequence, from the hairpin-loop structures (coordinate 1) onwards, of the DNA-B components isolated in this study. Duplicated sequences spanning the right (3′) leg of the stem loop structure (underlined) are highlighted in red. The 3′ repeat is the bona fide right (3′) leg of the components. The blue and orange boxes highlight sequences originating from BYVMV and CLCuMuV, respectively. (TIF) [file pone.0040050.s002.tif]

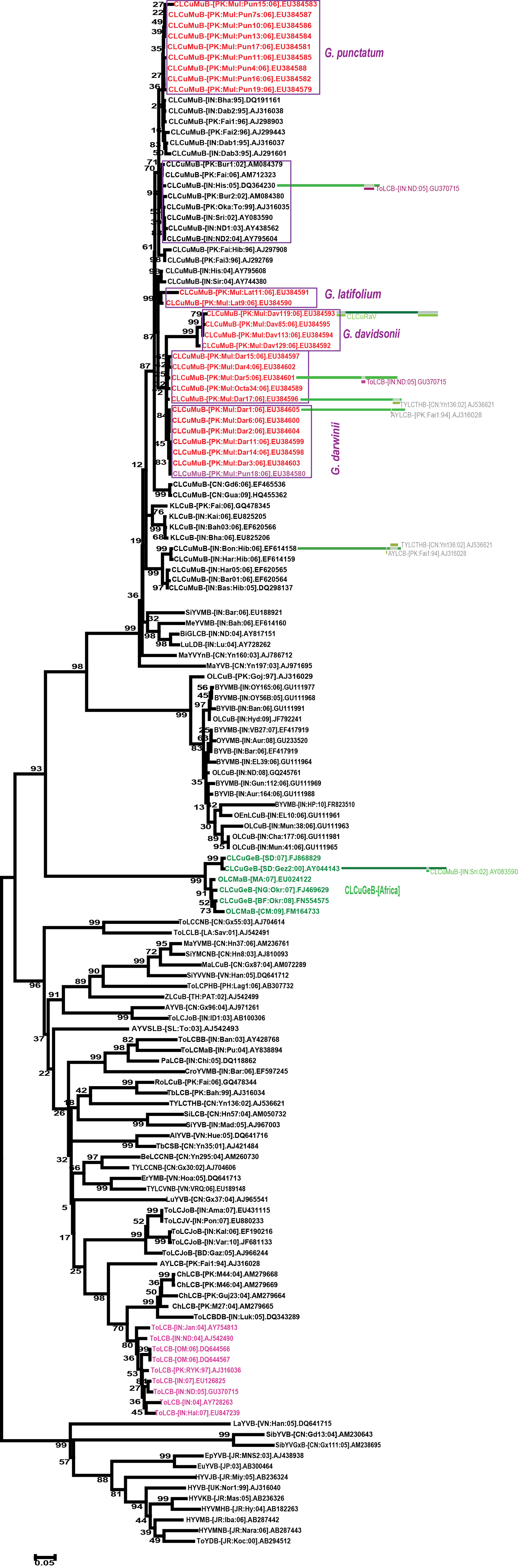

Supplement: Figure S3 — Phylogenetic analysis of betasatellite sequences. This tree differs from that in Figure 5 only in containing more sequences; sequences representing all so far identified betasatellite species. For other details about the tree see the figure legend of Figure 5. (TIF) [file pone.0040050.s003.tif]
