# Supplementary material for: A Melting Pot of Old World Begomoviruses and Their Satellites Infecting a Collection of Gossypium Species in Pakistan
Source: PLoS One. 2012 Aug 10;7(8):e40050. doi: 10.1371/journal.pone.0040050 (PMC3416816; doi:10.1371/journal.pone.0040050)
Supplement: Table S1 — List of virus genomes and genomic components isolated from Gossypium species. (DOC) [file pone.0040050.s004.doc]

**Table S1.** List of virus genomes and genomic components isolated from *Gossypium* species.

| Plant species | Species-  [isolate  descriptor] | Begomovirus | | | | | | | | DNA B | | | |
| --- | --- | --- | --- | --- | --- | --- | --- | --- | --- | --- | --- | --- | --- |
|  |  | Accession  number | Size  (nt) | Predicted open reading frames  (coding capacity [no. of amino acids]/ predicted molecular weight (kDa)/[start-stop codon coordinates]) | | | | | | Accession  number | Size  (nt) | Predicted open reading frames  (coding capacity [no. of amino acids]/ predicted molecular  weight (kDa)/[start-stop codon coordinates]) | |
|  |  |  |  | CP | V2 | Rep | TrAP | REn | C4 |  |  | NSP | MP |
| *G. punctatum* | GPMLCuV-  [PK:Mul:06] | EU384575 | 2737 | 257/29.60/  [283-1053] | 133/14.03/  [123-488] | 364/40.93/  [1502-2593] | 151/17.48/  [1153-1605] | 139/15.60  [1056-1460] | 86/9.34  [2179-2436] | EU384578 | 2673 | 260/29.83  [587-1366] | 274/31.11  [2202-1381] |
| *G. davidsonii* | CLCuRaV-  PK:Mul:Dav:06] | EU365616 | 2738 | 257/29.62/  [276-1046] | 133/14.01/  [116-481] | 363/41.02/  [1495-2583] | 151/17.30/  [1146-1598] | 139/15.66/  [1049-1453] | 101/11.00  [2127-2429] | - | - | - | - |
| *G. davidsonii* | GPMLCuV- [PK:Mul:Dav:06] | EU365617 | 2737 | 257/29.60/  [283-1053] | 133/14.03/  [123-488] | 364/40.92/  [1502-2593] | 151/17.48/  [1153-1605] | 139/15.60/  [1056-1460] | 86/9.34/  [2179-2436] | EU384577 | 2708 | 26129.74/  [590-1372] | 275/31.08/  [1381-2205] |
| *G. darwinii* | CLCuMuV- [PK:Mul:Dar1:06] | EU365613 | 2740 | 257/29.63/  [275-1045] | 133/14.00/  [115-480] | 363/41.01/  [1494-2582] | 151/16.67/  [1145-1597] | 139/15.77/  [1048-1452] | 101/11.37/  [2126-2428] | EU384576 | 2725 | 261/29.78/  [552-1334] | 274/31.08/  [1343-2164] |
| *G. darwinii* | CLCuMuV-  [PK:Mul:Dar3:06] | EU365614 | 2735 | 257/29.52/  [280-1050] | 133/14.04/  [120-485] | 363/41.01/  [1499-2587] | 151/17.33/  [1150-1602] | 139/15.52/  [1053-1457] | 101/11.34/  [2131-2433] | - | - | - | - |
| *G. darwinii* | CLCuMuV- [PK:Mul:Dar4:06] | EU365615 | 2739 | 257/29.61/  [275-1045] | 133/14.03/  [115-480] | 363/41.01/  [1497-2585] | 151/17.41/  [1145-1600] | 139/15.58/  [1048-1452] | 101/11.34/  [2129-2431] | - | - | - | - |
| *G. mustilinum* | CLCuRaV-  PK:Mul:Mus3:06] | EU384574 | 2736 | 257/29.00/  [276-1046] | 133/14.01/  [116-481] | 362/40.85/  [1495-2580] | 151/17.39/  [1146-1598] | 139/15.42/  [1049-1453] | 101/10.96/  [2124-2426] | FJ218489 | 2675 | 260/29.60/  [585-1364] | 275/31.12  [1373-2197] |
| *G. latifolium* | CLCuMuV-  [PK:Fai:Lat:06] | EU384573 | 2757 | 257/29.59/  [284-1054] | 133/14.31/  [121-489] | 364/40.82/  [1503-2594] | 151/17.50/  [1154-1606] | 139/15.71/  [1057-1461] | 101/11.33/  [2135-2437] | - | - | - | - |
| *G. hirsutum* | CLCuBuV-  [PK:Mul:Oct2:06] | EU365618 | 2762 | 257/29.63/  [292-1062] | 119/13.71/  [132-488] | 365/39.68/  [1505-2599] | 139/15.56/  [1204-1608] | 139/15.48/  [1059-1463] | 101/11.18/  [2140-2442] | - | - | - | - |
| *G. hirsutum* | CLCuBuV-  [PK:Mul:Oct5:06] | EU365619 | 2762 | 257/29.63/  [292-1062] | 119/13.71/  [132-488] | 365/39.68/  [1505-2599] | 139/15.56/  [1204-1608] | 139/15.48/  [1059-1463] | 101/11.16  [2140-2442] | - | - | - | - |
| *G. hirsutum* | CLCuBuV-  [PK:Mul:Oct7:06] | EU384570 | 2762 | 256/29.6/  [292-1062] | 118/13.7/  [132-485] | 364/41.0/  [2599-1508] | 134/15.6/  [1608-1207] | 134/15.5/  [1463-1062] | 181/20.4/  [2685-2143] | - | - | - | - |
| *G. hirsutum* | CLCuBuV [PK:Mul:Oct20:06] | EU384572 | 2762 | 257/29.63/  [292-1062] | 119/13.71/  [132-488] | 365/39.68/  [1505-2599] | 139/15.56/  [1204-1608] | 139/15.48/  [1059-1363] | 101/11.23/  [2140-2442] | - | - | - | - |
| *G. hirsutum* | CLCuMuV-  PK:Mul:Hir:08] | FJ218486 | 2735 | 255/29.48/  [281-1045] | 121/13.93/  [121-483] | 362/41.01/  [1494-2582] | 151/17.19/  [1145-1597] | 139/15.56/  [1048-1452] | 101/11.19/  [2126-2428] | - | - | - | - |
| *G. gossypioides* | GPMLCuV-  [PK:Mul:Gos:08] | FJ218485 | 2720 | 257/29.60/  [277-1047] | 122/14.03/  [117-482] | 363/41.70/  [1496-2587] | 151/17.48/  [1147-1599] | 139/15.60/  [1050-1454] | 91/9.57/  [2158-2430] | FJ218494 | 2681 | 261/29.89  [587-1369] | 275/31.12  [1378-2202] |
| *G. lobatum* | GPMLCuV-[PK:Mul:Lob1:08] | FJ210467 | 2729 | 257/29.91/  [281-1051] | 122/14.25/  [121-483] | 362/41.40/  [1500-2588] | 151/17.36/  [1151-1603] | 139/15.64/  [1054-1458] | 97/10.86/  [2141-2431] | FJ218488 | 2679 | 261/29.80/  [585-1367] | 275/31.11/  [1376-2200] |
| *G. somalense* | CLCuMuV-  [PK:Mul:Som:08] | FJ218487 | 2739 | 257/29.62/  [281-1051] | 123/13.98/  [121-486] | 350/37.80/  [1537-2586] | 152/16.87/  [1146-1601] | 139/15.66/  [1054-1458] | 101/11.28/  [2130-2432] | FJ218490 | 2771 | 260/29.68/  [581-1360] | 275/31.13/  [1369-2193] |
| *G. stocksii* | CLCuKoV-  [PK:Mul:Sto:08] | HM468427 | 2748 | 257/29.70/  [292-1062] | 119/13.74/  [132-488] | 361/40.31/  [1511-2593] | 139/15.29/  [1204-1608] | 139/15.65/  [1059-1463] | 101/11.08/  [2137-2439] | FJ218491 | 2766 | 258/29.52/  [522-1295] | 275/31.11/  [1304-2128] |
