# Supplementary material for: A Melting Pot of Old World Begomoviruses and Their Satellites Infecting a Collection of Gossypium Species in Pakistan
Source: PLoS One. 2012 Aug 10;7(8):e40050. doi: 10.1371/journal.pone.0040050 (PMC3416816; doi:10.1371/journal.pone.0040050)
Supplement: Table S2 — ACMV DNA-A clones isolated from cotton species. (DOC) [file pone.0040050.s005.doc]

**Table S2.** ACMV DNA-A clones isolated from cotton species.

| Isolate descriptor | Accession number | Host | Size  (nt) | Predicted open reading frames  (coding capacity [aa]/ predicted molecular weight [kDa]/[start-stop codon coordinates] | | | | | |
| --- | --- | --- | --- | --- | --- | --- | --- | --- | --- |
|  |  |  |  | AV1 | AV2 | AC1 | AC2 | AC3 | AC4 |
| [PK:Mul:Gos1:07] | FJ751235 | *G. gossypioides* | 2781 | 258/30.19/  [296-1069] | 113/13.11/  [136-474] | 358/40.26/  [2606-1533] | 135/15.09/  [1621-1217] | 134/15.89/  [1473-1072] | 112/12.50/  [2452-2117]* |
| [PK:Mul:Gos2:08] | GQ204106 | *G. gossypioides* | 2782 | 258/29.82/  [296-1069]@ | 113/13.11/  [136-474] | 358/40.38/  [2606-1533] | 135/15.09/  [1621-1217] | 134/15.89/  [1473-1072] | 140/15.47/  [2572-2153] |
| [PK:Mul:Har:08] | GQ204107 | *G. harknessii* | 2784 | 259/30.32/  [296-1072]^#^ | 113/13.11/  [136-474] | 358/40.40/  [2609-1536] | 135/15.06/  [1624-1220] | 134/15.89/  [1476-1075] | 140/15.37/  [2575-2156] |
| [PK:Mul:Lob1:08] | FJ751234 | *G. lobatum* | 2781 | 258/30.19/  [296-1069] | 113/13.11/  [136-474] | 358/40.60/  [2606-1533]^$^ | 135/14.95/  [1621-1217] ^$^ | 134/15.89/  [1473-1072] | 140/15.37/  [2572-2153] |
| [PK:Mul:Pun19:06] | FJ751233 | *G. punctatum* | 2756 | -^£^ | -^£^ | 358/40.37/  [2581-1508] | 135/15.09/  [1596-1192] | 134/15.88/  [1448-1047] | 140/15.31/  [2547-2128] |
| [PK:Mul:Som:08] | GQ204108 | *G. somalense* | 2781 | 258/30.17/  [296-1069] | 113/13.11/  [136-474] | 358/40.46/  [2606-1533]% | 135/15.09/  [1621-1217] | 134/15.89/  [1473-1072] | 140/15.37/  [2572-2153] |
| [PK:Mul:Sto:08] | GQ204109 | *G. stocksii* | 2781 | 258/30.19/  [296-1069] | 113/13.11/  [136-474] | 358/40.47/  [2606-1533] | 135/15.07/  [1621-1217] | 134/15.89/  [1473-1072] | 140/15.37/  [2572-2153] |
| [PK:Mul:Dar:06] | GQ169505 | *G. darwinii* | 2738 | -† | -† | 358/40.37/  [2563-1490] | 135/15.09/  [1578-1174] | 134/15.88/  [1430-1029] | 140/15.31/  [2529-2110] |
| [PK:Mul:Tom:08] | HM468428 | *G. tomentoseum* | 2789 | 259/30.43/  [296-1073] | 113/13.05/  [136-474] | 337/38.73/  [2612-1533] | 135/15.10/  [1624-1217] | 134/15.89/  [1476-1072] | - |

**Footnote to Table S2.**

*40 extra aa N-terminus but conserved methionine start codon.

@Deletion (of a T at 623) and insertion (of a T at 732) leading to replacement of 26aa with non cp sequence.

^#^ Insertion (of A at 727) and deletion (of A at 751), as well 3 further insertions (TA at 753 and T at 757) leading to replacement of 13aa with non cp sequence and the addition of one aa.

^$^15 aa near end of Rep (from 341 to 356 of Rep) due to frame shift deletion (deletion of C at 1583 and addition of G at 1537) that also affects AC2 near the N terminus.

^£^ The CP gene is replaced with a V2 coding sequence which has the potential to code for a 95aa V2 product. The *bona fide* V2 consists of the sequence encoding the first 51 aa, but with many sequence changes, fused to sequence encoding approximately 46 aa of CP sequence, again with many sequence changes. Between the two repeated V2 sequences lies approximately 470 nt of sequence of indeterminate origin.
