# Supplementary material for: A Melting Pot of Old World Begomoviruses and Their Satellites Infecting a Collection of Gossypium Species in Pakistan
Source: PLoS One. 2012 Aug 10;7(8):e40050. doi: 10.1371/journal.pone.0040050 (PMC3416816; doi:10.1371/journal.pone.0040050)
Supplement: Table S3 — Details of recombination between CGs detected using RDP3. (DOC) [file pone.0040050.s006.doc]

**Table S3.** Details of recombination between CGs detected using RDP3

| **Isolate name** | **Event** | **Average p value** |
| --- | --- | --- |
| CLCuBaV-[IN:Ban:04]AY705380 | a | 1.05 x 10^-08^ |
|  | b | 2.109 x 10^-09^ |
| CLCuBuV-[PK:Mul:Oct2:06]EU365618 | a | 1.78 x 10^-14^ |
|  | b | 2.867 x 10^-05^ |
| CLCuKoV-[IN:Dab:03]AY456683 | a | 1.778 x 10^-11^ |
|  | b | 1.468 x 10^-07^ |
| CLCuKoV[PK:Mul:Sto1:08]HM468427 | a | 1.778 x 10^-11^ |
|  | b | 1.468 x 10^-07^ |
| CLCuMuV-[IN:Lud:04]AY765257 | a | 4.393 x 10^-18^ |
|  | b | 4.307 x 10^-04^ |
| CLCuMuV-[PK:Hirs1:08]FJ218486 | a | 1.004 x 10^-05^ |
|  | b | 4.367 x 10^-16^ |
|  | c | 4.307 x 10^-04^ |
|  | d | 1.748 x 10^-05^ |
|  | e | 6.136 x 10^-04^ |
| CLCuMuV-[PK:Mul:Dar1:06]EU365613 | a | 4.627 x 10^-04^ |
| CLCuMuV-[PK:Mul:Dar3:06]EU365614 | a | 3.22 x 10^-03^ |
|  | b | 4.627 x 10^-04^ |
| CLCuMuV-[PK:Mul:Som:08]FJ218487 | a | 8.61 x 10^-05^ |
|  | b | 1.84 x 10^-09^ |
|  | c | 6.136 x 10^-04^ |
| CLCuRaV-[PK:Mul:Dav:06]EU365616 | a | 3.507 x 10^-06^ |
|  | b | 1.748 x 10^-11^ |
| CLCuRaV-[PK:Mul:Mus3:06]EU384574 | a | 3.121 x 10^-02^ |
|  | b | 1.748 x 10^-11^ |
| CLCuShV[PK:05]FN562001 | a | 1.295 x 10^-17^ |
|  | b | 1.788 x 10^-13^ |
|  | c | 1.318 x 10^-04^ |
| CLCuShV[PK:05]FN562001 | a | 1.295 x 10^-17^ |
|  | b | 1.788 x 10^-13^ |
|  | c | 1.318 x 10^-04^ |
| GPMLCuV[PK:Mul:06]EU384575 | a | 1.004 x 10^-05^ |
|  | b | 3.041 x 10^-18^ |
|  | c | 2.704 x 10^-05^ |
|  | d | 2.501 x 10^-23^ |
| GPMLCuV[PK:Mul:Dav:06]EU365617 | a | 1.004 x 10^-05^ |
|  | b | 3.041 x 10^-18^ |
|  | c | 2.704 x 10^-05^ |
|  | d | 2.501 x 10^-23^ |
| GPMLCuV[PK:Mul:Gos:08]FJ218485 | a | 5.464 x 10^-09^ |
|  | b | 3.041 x 10^-18^ |
|  | c | 2.704 x 10^-05^ |
|  | d | 2.501 x 10^-23^ |
| GPMLCuV[Pk:Mul:Lob:08]FJ210467 | a | 1.004 x 10^-05^ |
|  | b | 5.299 x 10^-17^ |
|  | c | 5.265 x 10^-09^ |
|  | d | 1.163 x 10^-07^ |
|  | e | 4.718 x 10^-18^ |

**Footnote for Table S3.**

Recombinant regions (a,b,c,d and e) are shown on figure 3. The average p-values of possible parental regions of recombinant viruses were identified through recombination detection program (RDP) embedded in RDP3. A cut off p-value 0.05 was used throughout the analysis.
