# Supplementary material for: A Melting Pot of Old World Begomoviruses and Their Satellites Infecting a Collection of Gossypium Species in Pakistan
Source: PLoS One. 2012 Aug 10;7(8):e40050. doi: 10.1371/journal.pone.0040050 (PMC3416816; doi:10.1371/journal.pone.0040050)
Supplement: Table S4 — CLCuMuB clones isolated from Gossypium species. (DOC) [file pone.0040050.s007.doc]

**Table S4.** CLCuMuB clones isolated from *Gossypium* species.

| **Isolate**  **descriptor** | **Accession**  **number** | **Host** | **Size (nt)** | **βC1**  **Coding capacity (amino acids)/**  **nucleotide coordinates/ predicted mol. weight (kDa)** |
| --- | --- | --- | --- | --- |
| [PK:Mul:Pun19:06] | EU384579 | *G. punctatum* | 1356 | 118/[195-551]/13.69 |
| [PK:Mul:Pun18:06] | EU384580 | *G. punctatum* | 1355 | 118/[195-551]/13.69 |
| [PK:Mul:Pun17:06] | EU384581 | *G. punctatum* | 1355 | 118/[195-551]/13.69 |
| [PK:Mul:Pun16:06] | EU384582 | *G. punctatum* | 1356 | 118/[195-551]/13.69 |
| [PK:Mul:Pun15:06] | EU384583 | *G. punctatum* | 1349 | 118/[188-544]/14.51 |
| [PK:Mul:Pun13:06] | EU384584 | *G. punctatum* | 1356 | 118/[195-551]/13.69 |
| [PK:Mul:Pun11:06] | EU384585 | *G. punctatum* | 1355 | 118/[195-551]/13.69 |
| [PK:Mul:Pun10:06] | EU384586 | *G. punctatum* | 1356 | 118/[195-551]/13.69 |
| [PK:Mul:Pun7s:06] | EU384587 | *G. punctatum* | 1356 | 118/[196-552]/13.69 |
| [PK:Mul:Pun4:06] | EU384588 | *G. punctatum* | 1355 | 118/[195-551]/13.69 |
| [PK:Mul:Octa34:06] | EU384589 | *G. hirsutum* | 1370 | 99/[195-494]/11.58 |
| [PK:Mul:Lat9:06] | EU384590 | *G. latifolium* | 1350 | 118/[195-551]/13.63 |
| [PK:Mul:Lat11:06]@ | EU384591 | *G. latifolium* | 1141 | 99/[195-494]/11.57 |
| [PK:Mul:Dav129:06]* | EU384592 | *G. davidsonii* | 1349 | 117/[201-554]/13.55 |
| [PK:Mul:Dav119:06]* | EU384593 | *G. davidsonii* | 1357 | 99/[201-500]/11.53 |
| [PK:Mul:Dav113:06]* | EU384594 | *G. davidsonii* | 1359 | 99/[201-500]/14.03 |
| [PK:Mul:Dav85:06]* | EU384595 | *G. davidsonii* | 1357 | 99/[201-500]/11.57 |
| [PK:Mul:Dar17:06] | EU384596 | *G. darwinii* | 1356 | 118/[195-551]/13.69 |
| [PK:Mul:Dar15:06] | EU384597 | *G. darwinii* | 1350 | 118/[195-551]/13.67 |
| [PK:Mul:Dar14:06] | EU384598 | *G. darwinii* | 1352 | 118/[194-550]/13.69 |
| [PK:Mul:Dar11:06] | EU384599 | *G. darwinii* | 1353 | 118/[195-551]/13.69 |
| [PK:Mul:Dar6:06] | EU384600 | *G. darwinii* | 1355 | 118/[195-551]/13.69 |
| [PK:Mul:Dar5:06] | EU384601 | *G. darwinii* | 1355 | 118/[195-551]/13.69 |
| [PK:Mul:Dar4:06] | EU384602 | *G. darwinii* | 1355 | 118/[195-551]/13.69 |
| [PK:Mul:Dar3:06] | EU384603 | *G, darwinii* | 1355 | 118/[195-551]/13.69 |
| [PK:Mul:Dar2:06] | EU384604 | *G. darwinii* | 1355 | 118/[195-551]/13.69 |
| [PK:Mul:Dar1:06] | EU384605 | *G. darwinii* | 1355 | 118/[195-551]/13.69 |

**Footnotes to Table S4**

*These molecules are recombinant, containing the origin of replication derived from a begomovirus and are thus, by convention, not classified as betasatellites.

@ Contains an inverted repeat of the βC1 coding sequence
