# Supplementary material for: A Melting Pot of Old World Begomoviruses and Their Satellites Infecting a Collection of Gossypium Species in Pakistan
Source: PLoS One. 2012 Aug 10;7(8):e40050. doi: 10.1371/journal.pone.0040050 (PMC3416816; doi:10.1371/journal.pone.0040050)
Supplement: Table S5 — Alphasatellites isolated from Gossypium species. (DOC) [file pone.0040050.s008.doc]

**Table S5.** Alphasatellites isolated from *Gossypium* species.

| **Species** | **Isolate descriptor** | **Accession number** | **Host** | **Size**  **(nt)** | **Rep**  **Coding capacity (amino acids)/**  **nucleotide coordinates/ predicted mol. weight (kDa)** |
| --- | --- | --- | --- | --- | --- |
| GDarSLA | [PK:Mul:Dar1:06] | EU384606 | *G. darwinii* | 1373 | 315/[70-1017]/36.60 |
|  | [PK:Mul:Dar2:06] | EU384607 | *G. darwinii* | 1369 | 315/[70-1017]/36.60 |
|  | [PK:Mul:Dar3:06] | EU384608 | *G. darwinii* | 1368 | 315/[70-1017]/36.34 |
|  | [PK:Mul:Dar4:06] | EU384609 | *G. darwinii* | 1368 | 315/[70-1017]/36.60 |
|  | [PK:Mul:Dar5:06] | EU384610 | *G. darwinii* | 1368 | 315/[70-1017]/36.43 |
|  | [PK:Mul:Dar6:06] | EU384611 | *G. darwinii* | 1373 | 315/[70-1017]/36.60 |
|  | [PK:Mul:Dar7:06] | EU384612 | *G. darwinii* | 1369 | 315/[70-1017]/36.60 |
|  | [PK:Mul:Dar7b:06] | EU384613 | *G. darwinii* | 1369 | 315/[70-1017]/36.60 |
|  | [PK:Mul:Dar9:06] | EU384614 | *G. darwinii* | 1368 | 315/[70-1017]/36.05 |
|  | [PK:Mul:Dar10:06] | EU384615 | *G. darwinii* | 1368 | 315/[70-1017]/36.02 |
|  | [PK:Mul:Dar11:06] | EU384616 | *G. darwinii* | 1367 | 315/[70-1017]/36.62 |
|  | [PK:Mul:Dar12:06] | EU384617 | *G. darwinii* | 1368 | 315/[70-1017]/36.78 |
|  | [PK:Mul:Dar13:06] | EU384618 | *G. darwinii* | 1369 | 315/[70-1017]/36.60 |
|  | [PK:Mul:Dar15:06] | EU384619 | *G. darwinii* | 1369 | 315/[70-1017]/36.50 |
|  | [PK:Mul:Dar17:06] | EU384620 | *G. darwinii* | 1368 | 315/[70-1017]/36.60 |
|  | [PK:Mul:Dar19:06] | EU384621 | *G. darwinii* | 1370 | 315/[70-1017]/36.50 |
|  | [PK:Mul:Dar20:06] | EU384622 | *G. darwinii* | 1368 | 315/[70-1017]/36.60 |
|  | [PK:Mul:Dav7C:06] | EU384623 | *G. davidsonii* | 1370 | 315/[70-1017]/36.56 |
|  | [PK:Mul:Dav8:06] | EU384624 | *G. davidsonii* | 1360 | 315/[70-1017]/36.56 |
|  | [PK:Mul:Dav12:06] | EU384625 | *G. davidsonii* | 1363 | 315/[70-1017]/36.58 |
|  | [PK:Mul:Dav13:06] | EU384626 | *G. davidsonii* | 1363 | 315/[70-1017]/36.56 |
|  | [PK:Mul:Dav16:06] | EU384627 | *G. davidsonii* | 1346 | 315/[70-1017]/36.56 |
|  | [PK:Mul:Dav19:06] | EU384628 | *G. davidsonii* | 1366 | 315/[70-1017]/36.56 |
|  | [PK:Mul:Dav21:06] | EU384629 | *G. davidsonii* | 1366 | 315/[70-1017]/36.56 |
|  | [PK:Mul:Dav22:06] | EU384630 | *G. davidsonii* | 1366 | 315/[70-1017]/36.56 |
|  | [PK:Mul:Mus6:06] | EU384631 | *G. mustilinum* | 1312 | 315/[70-1017]/36.59 |
|  | [PK:Mul:MusC4D8:06] | EU384632 | *G. mustilinum* | 1365 | 315/[70-1017]/36.56 |
|  | [PK:Mul:MusD3E7:06] | EU384633 | *G. mustilinum* | 1365 | 315/[70-1017]/36.56 |
|  | [PK:Mul:MusE3F7:06] | EU384634 | *G. mustilinum* | 1365 | 315/[70-1017]/36.56 |
|  | [PK:Mul:MusH3G7:06] | EU384635 | *G. mustilinum* | 1310 | 315/[47-994/36.56 |
|  | [PK:Mul:Oct3:06] | EU384636 | *G. hirsutum* | 1362 | 315/[70-1017]/36.56 |
|  | [PK:Mul:Oct4:06] | EU384637 | *G. hirsutum* | 1376 | 315/[70-1017]/36.51 |
|  | [PK:Mul:Oct9:06] | EU384638 | *G. hirsutum* | 1369 | 315/[70-1017]/36.64 |
|  | [PK:Mul:Oct10:06] | EU384639 | *G. hirsutum* | 1359 | 315/[70-1017]/36.17 |
|  | [PK:Mul:Oct11:06] | EU384640 | *G. hirsutum* | 1370 | 315/[70-1023]/36.17 |
|  | [PK:Mul:Oct12:06] | EU384641 | *G. hirsutum* | 1378 | 317/[70-1023]/36.95 |
|  | [PK:Mul:Oct15:06] | EU384642 | *G. hirstutum* | 1371 | 315/[70-1017]/36.35 |
|  | [PK:Mul:Oct21:06] | EU384643 | *G, hirsutum* | 1372 | 315/[70-1017]/36.22 |
|  | [PK:Mul:Oct28:06] | EU384644 | *G, hirsutum* | 1367 | 315/[70-1017]/36.30 |
|  | [PK:Mul:Pun2:06] | EU384645 | *G. punctatum* | 1377 | 315/[70-1017]/36.27 |
|  | [PK:Mul:Pun3:06] | EU384646 | *G. punctatum* | 1372 | 315/[70-1017]/36.17 |
|  | [PK:Mul:Pun5:06] | EU384647 | *G. punctatum* | 1376 | 315/[69-1016]/35.62 |
|  | [PK:Mul:Pun8:06] | EU384648 | *G. punctatum* | 1358 | 315/[69-101]/36.63 |
|  | [PK:Mul:Pun11:06] | EU384649 | *G. punctatum* | 1365 | 315/[70-1016]/36.56 |
|  | [PK:Mul:Pun15:06] | EU384651 | *G. punctatum* | 1363 | 315/[69-1019]/36.89 |
|  | [PK:Mul:Pun16:06] | EU384652 | *G. punctatum* | 1363 | 315/[70-1017]/36.11 |
| GMusSLA | [PK:Mul:Gos-2:08] | FJ218494 | *G. gossypoidies* | 1363 | 295/[59-964]/34.51 |
|  | [PK:Mul:Lat1:08] | FJ218495 | *G. latifolium* | 1386 | 302/[72-980]/35.18 |
|  | [PK:Mul:Lob-3:08] | FJ218496 | *G. lobatum* | 1362 | 295/[59-946]/34.51 |
|  | [PK:Mul:Mus1B:06] | EU384655 | *G. mustilinum* | 1315 | 295/[59-946]/34.48 |
|  | [PK:Mul:Mus2:06] | EU384656 | *G. mustilinum* | 1306 | 295/[59-946]/34.48 |
|  | [PK:Mul:Mus3:06] | EU384657 | *G. mustilinum* | 1309 | 295/[59-946]/34.48 |
|  | [PK:Mul:Mus3B:06] | EU384658 | *G. mustilinum* | 1321 | 295/[59-946]/34.48 |
|  | [PK:Mul:Mus4:06] | EU384659 | *G. mustilinum* | 1307 | 295/[59-946]/34.47 |
|  | [PK:Mul:MusF3G7:06] | EU384660 | *G. mustilinum* | 1309 | 295/[59-946]/34.48 |
|  | [PK:Mul:MusH3A8:06] | EU384661 | *G. mustilinum* | 1321 | 295/[59-946]/34.48 |
|  | [PK:Mul:Mus1C:06] | EU384662 | *G. mustilinum* | 1315 | 295/[59-946]/34.48 |
|  | [PK:Mul:Pun9:06] | EU384663 | *G. punctatum* | 1318 | 295/[58-945]/34.17 |
|  | [PK:Mul:Dav7B:06] | EU384653 | *G. davidsonii* | 1218 | 263*/[58-849]/30.79 |
|  | [PK:Mul:Mus1:06] | EU384654 | *G. mustilinum* | 1218 | 263*/[58-849]/30.79 |

**Footnote to Table S5.**

*Rep gene truncated due to frame shift mutation at the C-terminal end introducing a premature stop codon. The mutation truncates the product by 34aa and introduces 2 additional non-Rep amino acids.
