# Supplementary material for: A Melting Pot of Old World Begomoviruses and Their Satellites Infecting a Collection of Gossypium Species in Pakistan
Source: PLoS One. 2012 Aug 10;7(8):e40050. doi: 10.1371/journal.pone.0040050 (PMC3416816; doi:10.1371/journal.pone.0040050)
Supplement: Table S6 — Upper and lower bounds of the 95% highest posterior density (HPD) estimates for divergence dates of CLCuMuV-encoded CP, CLCuMuB-encoded βC1 and GDarSLA-encoded Rep. (DOC) [file pone.0040050.s009.doc]

**Table S6.** Upper and lower bounds of the 95% highest posterior density (HPD) estimates for divergence dates of CLCuMuV-encoded CP, CLCuMuB-encoded βC1 and GDarSLA-encoded Rep

| Gene | Node | Mean value | 95% HPDs |
| --- | --- | --- | --- |
| CP | Base | 1755 | 1803-684 |
|  | A | 1887 | 1929-1491 |
|  | B | 1872 | 1900-1370 |
|  | C | 1987 | 1994-1919 |
|  | D | 1966 | 1979-1830 |
|  | E | 1976 | 1990-1876 |
|  | F | 1928 | 1951-1614 |
|  | G | 1935 | 1956-1657 |
| βC1 | Base | 1653 | 1691-781 |
|  | A | 1966 | 1989-1892 |
|  | B | 1931 | 1961-1792 |
|  | C | 1952 | 1967-1835 |
|  | D | 1945 | 1968-1835 |
|  | E | 1963 | 1984-1890 |
| Rep | Base | 1892 | 1928-1631 |
|  | A | 1968 | 1987-1905 |
|  | B | 1958 | - |
|  | C | 1961 | 1979-1890 |
|  | D | 1997 | 2003-1969 |
|  | E | 1983 | 1997-1946 |
|  | F | 1996 | 2002-1987 |
